# Fatostatin induces ferroptosis through inhibition of the AKT/mTORC1/GPX4 signaling pathway in glioblastoma

**DOI:** 10.1038/s41419-023-05738-8

**Published:** 2023-03-25

**Authors:** Jiayang Cai, Zhang Ye, Yuanyuan Hu, Liguo Ye, Lun Gao, Yixuan Wang, Qian sun, Shiao Tong, Shenqi Zhang, Liquan Wu, Ji’an Yang, Qianxue Chen

**Affiliations:** 1 Department of Neurosurgery, Renmin Hospital of Wuhan University, 430060 Wuhan, Hubei China; 2 Central Laboratory, Renmin Hospital of Wuhan University, 430060 Wuhan, Hubei China; 3 Department of Ophthalmology, Tongji Hospital, Tongji Medical College, Huazhong University of Science and Technology, 430030 Wuhan, China

**Keywords:** CNS cancer, Drug development, Drug delivery, Cell death

## Abstract

Glioblastoma multiforme (GBM) is the most common and fatal primary malignant central nervous system tumor in adults. Although there are multiple treatments, the median survival of GBM patients is unsatisfactory, which has prompted us to continuously investigate new therapeutic strategies, including new drugs and drug delivery approaches. Ferroptosis, a kind of regulated cell death (RCD), has been shown to be dysregulated in various tumors, including GBM. Fatostatin, a specific inhibitor of sterol regulatory element binding proteins (SREBPs), is involved in lipid and cholesterol synthesis and has antitumor effects in a variety of tumors. However, the effect of fatostatin has not been explored in the field of ferroptosis or GBM. In our study, through transcriptome sequencing, in vivo experiments, and in vitro experiments, we found that fatostatin induces ferroptosis by inhibiting the AKT/mTORC1/GPX4 signaling pathway in glioblastoma. In addition, fatostatin inhibits cell proliferation and the EMT process through the AKT/mTORC1 signaling pathway. We also designed a p28-functionalized PLGA nanoparticle loaded with fatostatin, which could better cross the blood-brain barrier (BBB) and be targeted to GBM. Our research identified the unprecedented effects of fatostatin in GBM and presented a novel drug-targeted delivery vehicle capable of penetrating the BBB in GBM.

## Introduction

Glioblastoma multiforme (GBM) is the most common and the most fatal primary malignant central nervous system tumor in adults, accounting for approximately half of all gliomas [1]. Evolving treatments, including surgical resection, TMZ chemotherapy, radiation therapy and immunotherapy, only maintain the median survival of GBM patients at 14.6 months [2]. Hence, the identification of novel therapeutic strategies and antitumor drugs is urgently needed to overcome this dilemma.

Ferroptosis has been defined as an iron-dependent regulated cell death (RCD) controlled by glutathione peroxidase 4 (GPX4) by the Nomenclature Committee on Cell Death (NCCD); this process is mainly initiated by severe lipid peroxidation due to ROS generation and iron availability [3, 4]. Accumulating evidence shows that ferroptosis plays a crucial role in human cancers, and several drugs can be used to treat tumors by targeting regulatory molecules of ferroptosis [5, 6]. In GBM, RSL3 was reported to drive ferroptosis by targeting GPX4 expression [7]. Su et al. showed that roxadustat can induce ferroptosis in chemoresistant GBM cells [8]. GBM is highly aggressive, and epithelial–mesenchymal transition (EMT) processes are thought to play a crucial role in the spread and metastasis of GBM [9]. Moreover, only a few therapeutic drugs, including gambogenic acid, β-elemene, and cetuximab, have been shown to induce ferroptosis while inhibiting EMT in tumor cells [10]. Therefore, an intensive study of the mechanisms of ferroptosis and EMT in GBM and the search for appropriate target drugs are important to improve the prognosis of GBM patients.

Fatostatin, a specific inhibitor of sterol regulator element binding proteins (SREBPs) that controls the expression of multiple key enzymes in the lipid and cholesterol synthesis pathways, inhibits SREBP maturation by blocking the translocation of SCAP from the endoplasmic reticulum to the Golgi apparatus [11]. Various studies have shown that fatostatin exerts an antitumor effect on several cancers through SREBP-dependent processes but also has many non-SREBP-dependent activities [12]. Most previous studies have focused on the role of fatostatin in inhibiting SREBPs followed by regulating cell proliferation, apoptosis, and the cell cycle in tumors, and few studies have investigated its relationship with ferroptosis, which is characterized by lipid peroxidation. In addition, due to the blood‒brain barrier (BBB), it is difficult for antitumor drugs to efficiently reach intracranial tumors to exert satisfactory therapeutic effects [13]. The application of nanotechnology facilitates the intracranial delivery of drugs and shows promise in overcoming the dilemma posed by the BBB [14–16]. Polylactic-co-glycolic acid (PLGA), a polymer composed of polyglycolic acid and polylactic acid, is widely applied in the pharmaceutical industry and certified by the Food and Drug Administration (FDA). This molecule has excellent biocompatibility, can be self-assembled into nanoparticles and is easily modified for targeted drug delivery [17]. PLGA degrades in the human physical environment to lactic and glycolic acids, which are products of human cell metabolism [18]. The peptide p28 is a kind of cell-penetrating peptide (CPP) that has potential to deliver therapeutic molecules to unreachable intracellular targets. As previously reported, p28-functionalized PLGA has been used to treat lung cancer by loading with gefitinib [19].

In this study, we first found that fatostatin could induce cell death and inhibit cell proliferation in GBM cell lines. To further investigate the inhibitory effects of fatostatin on GBM, we performed transcriptome sequencing and found that fatostatin is involved in ferroptosis, apoptosis, EMT, and several signaling pathways in GBM cells. By further validation, we found that fatostatin could inhibit the EMT process of GBM. Fatostatin-induced GPX4-mediated ferroptosis but not apoptosis. Regarding the molecular mechanism, we found that fatostatin induces ferroptosis by inhibiting GPX4 synthesis through inhibition of the AKT/mTORC1 signaling pathway. To better target GBM, we designed p28-functionalized PLGA nanoparticles loaded with fatostatin, which could better cross the BBB and be targeted for delivery to GBM. Our research identified the unprecedented effects of fatostatin in GBM and presented a novel drug-targeted delivery vehicle capable of penetrating the BBB in GBM.

## Materials and methods

### Antibodies and reagents

The antibodies included the following: anti-E-cadherin (20874-1-AP, Proteintech, Wuhan), anti-N-cadherin (22018-1-AP, Proteintech, Wuhan), anti-SNAI1 (13099-1-AP, Proteintech, Wuhan), anti-Vimentin (10366-1-AP, Proteintech, Wuhan), anti-GPX4 (A1933, Abclonal, Wuhan), anti-β-tubulin (M20005, Abmart, Shanghai), anti-AKT (60203-2-Ig, Proteintech, Wuhan), anti- phospho-AKT (Thr308) (29163-1-AP, Proteintech, Wuhan), anti-mTOR (66888-1-Ig, Proteintech, Wuhan), anti-phospho-mTOR (67778-1-Ig, Proteintech, Wuhan), anti- eIF4EBP1 (A19045, Abclonal, Wuhan), anti-phospho-eIF4EBP1 (AP0030, Abclonal, Wuhan), anti-Ki67 (GB121141, Servicebio, Wuhan), anti-SLC7A11 (26864-1-AP, Proteintech, Wuhan), anti-ACSL4 (A6826, Abclonal, Wuhan), and anti-FTL (ab69090, Abcam, UK). The AKT activator SC79 (HY-18749) and mTOR activator MHY1485 (HY-B0795) were purchased from MCE (USA). P28 peptide (sequence: LSTAADMQGVVTDGMASGLDKDYLKPDDC) was purchased from GenScript. Chloroquine (CQ), deferoxamine (DFO), and the proteasome inhibitor MG132 were purchased from MCE. The apoptosis inhibitor Z-VAD-FMK (S7023), the ferroptosis inhibitor ferrostatin-1 (S7243), and the necrosis inhibitor necrosulfonamide (S8251) were purchased from Selleck (USA). Cycloheximide was obtained from Sigma‐Aldrich.

### Cell culture

We purchased human GBM cell lines (U87 and U251) from the Cell Bank of the Shanghai Institute of Biochemistry and Cell Biology (Shanghai, China). High-glucose DMEM (Genom, Hangzhou, China) containing 10% fetal bovine serum (Thermo Fisher Scientific) and 1% penicillin/streptomycin (Biosharp, Anhui, China) was used to culture the cells, and the incubation temperature was 37 °C with 5% CO_2_.

### Cell viability assay

Cell Counting Kit-8 (CCK-8) (Topscience, Shanghai, China) was used to measure cell viability. We seeded 5000 cells per well in 96-well plates and treated them with different concentrations of fatostatin and other reagents for 24 h. According to the protocol provided by the supplier, we added 10 μl per well of CCK-8 regents and incubated it for 1 h at 37 °C. One hour later, a Multimode Plate Reader (PerkinElmer, Germany) was used to detect the absorbance value at 450 nm.

### Live/dead cell double staining assay

The cells were seeded in a 96-well plate and treated with DMSO or 20 μM fatostatin for 24 h. Then, according to the instructions of the reagent manufacturer, 100 μl of calcein AM/PI detection working solution was added to each well and incubated at 37 °C for 30 min in the dark. Finally, a fluorescence microscope (Olympus IX71, Japan) was used to capture images.

### Colony-formation assay

For colony-formation assays, six-well plates were seeded with 500 cells per well, and the cells were cultured for approximately two weeks. Four percent paraformaldehyde (Biosharp, Anhui, China) was used to fix the colonies, and 0.1% crystal violet (G1014, Servicebio, Wuhan) was used for staining. Then, representative colonies were captured and quantified.

### EdU-DNA synthesis assay

An EdU Cell Proliferation Kit with Alexa Fluor 555 (MA0425, Meilun, Dalian) was used to detect proliferating cells after treatment with different concentrations of fatostatin for 24 h. The prewarmed EdU working solution was added to the treated cells for EdU labeling for 2 h. After removal of the medium, the cells were fixed for 30 min, and then, 500 μl of Click reaction solution was added to each well and incubated at room temperature for 30 min in the dark. Finally, 1 ml of Hoechst 33342 solution was added to each well, and the cells were incubated at room temperature for 10 min in the dark. After staining, a fluorescence microscope (Olympus BX51, Japan) was used to take the image.

### RNA-Seq

Total RNA was extracted using TRIzol reagent (Invitrogen) after cells were treated with different concentrations of fatostatin for 24 h. A NovaSeq 6000 instrument and NovaSeq S4 reagent kit were used to perform sequencing. Differentially expressed gene (DEG) analysis of two groups was performed using the DESeq R package (1.18.1), and |log2(FoldChange)| > 1 and adjusted *P* value < 0.05 were set as criteria. Gene Ontology (GO), KEGG, GSEA, and Reactome enrichment analyses between two groups were implemented by the R package “clusterProfiler”, and genes with adjusted *P* values or *P* values less than 0.05 were considered significantly enriched.

### Wound-healing assay

A 200 μL pipette tip was used to scrape the cells in 6-well plates to produce wounds. Medium containing only 1% FBS was used to culture the cells to exclude the effect of cell proliferation. After the cells were treated with different concentrations of fatostatin, the wound area was photographed at 0 h, 24 h, and 48 h. Representative boundaries of the wound were drawn with dotted lines. Wound size was quantified by ImageJ (version 1.53e). The wound-healing percentage was calculated and analyzed.

### Transwell assay

Transwell chambers and polycarbonate membranes coated with Matrigel (R&D, USA) were used to perform the Transwell assay. The cells treated with different concentrations of fatostatin or other reagents were seeded in the upper chamber with 200 µl of serum-free medium, and 600 µl of medium with 10% FBS was added into the lower chamber as a chemoattractant. After incubation of the cells in an incubator at 37 °C and 5% CO_2_ for 24 h and fixation with 4% paraformaldehyde, 0.5% crystal violet was used to stain the cells on the lower side of the Transwell chamber. An inverted microscope (Olympus BX51, Japan) was used to take the image and record the cell number.

### Western blot analysis

Cells were lysed according to the method described previously to obtain processed protein [20]. Then, we separated the processed proteins by SDS‒PAGE and transferred them to a PVDF membrane (Millipore, Germany). After the PVDF membrane was blocked, the membrane was successively incubated with the diluted primary antibody solution and the secondary antibody (Proteintech, Wuhan, China). A ChemiDoc Touch (Bio-Rad, USA) was used to capture the image. The relative protein level was normalized to β-tubulin.

### Detection of lipid peroxidation level

Intracellular malondialdehyde (MDA) levels in GBM cells were detected by a Lipid Peroxidation MDA Assay Kit (S0131S, Beyotime, China). Total protein was detected by a BCA Kit (P0012, Beyotime) and used to calculate the relative MDA concentration. Lipid ROS were labeled with 2 μM C11-BODIPY 581/591 (Thermo Fisher, USA) for 30 min and detected by a FACSCalibur flow cytometer (Becton Dickinson). FlowJo X (Version 10.0.7) software was used to analyze the results.

### Glutathione assays

A Glutathione Assay Kit (S0053, Beyotime) was used to detect the GSH concentration in GBM cells. Total protein was detected by a BCA Kit (P0012, Beyotime) and used to calculate the relative GSH concentration.

### Transmission electron microscopy (TEM)

Cell samples were collected and fixed in a fixation solution containing 2.5% glutaraldehyde (Servicebio). Then, 1% osmium tetroxide and dehydration were used for postfixation. After embedding the samples in Epon, we stained the specimen sections with uranyl acetate. A transmission electron microscope was used to capture the pictures (Hitachi HT7700, Tokyo, Japan).

### Quantitative real-time PCR

After the extraction of RNA from GBM cells, we synthesized cDNA with the PrimeScript RT Reagent Kit (RR047A, TaKaRa, Japan). SYBR Premix Ex Taq II (RR820A, TaKaRa, Kusatsu, Japan) and Bio-Rad CFX Manager 2.1 real-time PCR systems (Bio-Rad, Hercules, CA, USA) were used to detect mRNA levels. The comparative Ct method was used to evaluate mRNA expression, and β-actin was used as an internal control.

### Scanning electron microscopy (SEM)

We used SEM to detect the morphology of the NPs. On a silicon wafer, 1 mg/ml NPs was added dropwise, followed by coating with gold for 60 s in a sputterer with a current of 40 mA. A field-emission scanning electron microscope (Zeiss GeminiSEM 500, Oberkochen, Germany) was used to capture the images.

### Preparation of p28-functionalized PLGA nanoparticles

p28-functionalized PLGA NPs were prepared using standard emulsion procedures [10]. We dissolved 10 mg PLGA, 1 mg PLGA-PEG-MAL, and 1 mg fatostatin together in a mixture of methanol (0.1 ml) and DCM (2 ml) (oil phase). After dropwise addition to a solution of 5 ml of 2.5% PVA (aqueous phase), we sonicated the resulting emulsion for 120 seconds on ice and added it dropwise to 40 ml of 0.3% PVA solution. We collected the NPs by centrifugation at 12000 rpm for 30 min after evaporation overnight at 4 °C. Then, we removed residual PVA by resuspending it in 50 ml of water and obtained the NPs by centrifugation at 12000 rpm for 30 min. Finally, we obtained purified aqueous solutions of fatostatin-loaded PLGA NPs with maleimide moiety surface display (NPs-FAT) after resuspending NPs in 1 ml of deionized water and sonicating for 120 s. Next, maleimide-thiol click chemistry was used to conjugate the p28 peptide to the NPs-FAT. We incubated p28 (3 mg) aqueous solution with TCEP (0.3 mg) for one hour at room temperature, added it to the aqueous solution of NPs-FAT (30 mg), gently magnetically stirred at 4 °C overnight (360 rpm) and centrifuged at 12000 rpm for 30 min. The purified aqueous solution of p28-PLGA NPs loaded with fatostatin (p28-NPs-FAT) was obtained after resuspending the precipitated particles in 1 ml of deionized water. We synthesized p28-NPs loaded with IR780 and coumarin-6 by the same method.

### Dynamic light scattering (DLS)

We used DLS to detect the hydrodynamic size and ζ-potential of the NPs. One milligram of NPs dissolved in ddH_2_O water was measured by a Malvern Zetasizer (Zetasizer Nano ZSP, Malvern, UK).

### Drug loading and release study

We used HPLC (Agilent 1100, Agilent, USA) to characterize drug loading. NPs were dissolved in DMSO to release fatostatin, and fatostatin was quantified by HPLC (Agilent 1100, Agilent, USA). To characterize drug release, we placed p28-NPs-FAT in a dialysis bag (MWCO 3000) against PBS and then immersed it in a preparation tube loaded with 40 mL of PBS, which was kept at 37 °C and shaken at a rate of 120 times/min. Then, we removed 1 mL of solution outside the dialysis bag for quantification by HPLC (Agilent 1100, Agilent, USA) at predetermined time intervals (0, 1, 2, 4, 6, 8, 16, 24, 48, and 72 h) and replaced it with the same volume of PBS. We calculated and plotted the cumulative release of fatostatin over time.

### HPLC

An Agilent 1100 (Agilent Technologies, Santa Clara, USA) system was used to carry out HPLC. Methanol or water was used as the eluent. Then, we recorded and processed the data with software.

### Cellular uptake

PLGA NPs and p28-PLGA NPs encapsulated with coumarin-6 (C6) were synthesized to characterize the uptake of NPs by GBM cells. The U87 cells were incubated with the NPs in an incubator at 37 °C and 5% CO2 for one hour. After fixation of the cells with paraformaldehyde, an Olympus BX53 microscope (Olympus) was used to observe cellular uptake.

### Intracranial xenograft model and imaging

All 6-week-old BALB/c nude mice were obtained from Shaulaibao Biotechnology Co., Ltd. (Wuhan, China). The Committee of Animal Care and Use of Renmin Hospital of Wuhan University approved all experiments with animals in this study. After anesthetizing the nude mice with isoflurane inhalation, we injected 1 × 10^6^ U87 cells that were engineered for the expression of luciferase into the right striatum (3.5 mm from the midline of the brain and 2 mm in front of the coronal suture, injection depth of 3 mm from the brain surface) of the nude mice to establish an intracranial xenograft model. For the detection of pharmacokinetics in mice, RhoB-loaded p28-PLGA NPs were injected into the mice (*n* = 3) through the tail vein. We collected blood samples at predetermined time points, quantified the RhoB concentrations, and plotted them with time. To characterize NPs for GBM treatment, we randomly divided the tumor-bearing mice into four groups (*n* = 8) treated with PBS, free fatostatin (25 mg/kg), NPs-FAT (fatostatin equivalent dose at 25 mg/kg), and p28-NPs-FAT (fatostatin equivalent dose at 25 mg/kg). After 7 days of tumor inoculation, the treatment was conducted 3 days per week for 4 weeks. In addition, we performed IVIS imaging of intracranial tumors at 1, 3, and 5 weeks after tumor inoculation to observe tumor progression. IVIS was also used to carry out imaging of IR780-loaded NPs. The mice were monitored regularly and euthanized when they exhibited severe neurological symptoms and/or obvious weight loss (>20% of their body weight). We sacrificed a separate cohort of mice five weeks after tumor inoculation for pathological staining (*n* = 3). The mouse brains were harvested, fixed, embedded, and sectioned for immunohistochemistry and H&E staining as described in our previous study [20].

### Statistical analysis

All the experimental assays in this study were repeated at least three times, and the data are presented as the mean ± standard deviation (SD). Unpaired Student’s *t* test was used to compare two groups of means. One-way ANOVA was used for comparisons among the different groups. Kaplan–Meier analysis (log-rank test) was used to detect the survival differences of nude mice. All statistical analyses were conducted by using GraphPad Prism 8. *P < 0.05, **P < 0.01, and ***P < 0.001 were regarded as statistically significant.

## Results

### Fatostatin induces cell death and inhibits cell proliferation in GBM cells

**Fig. 1:**
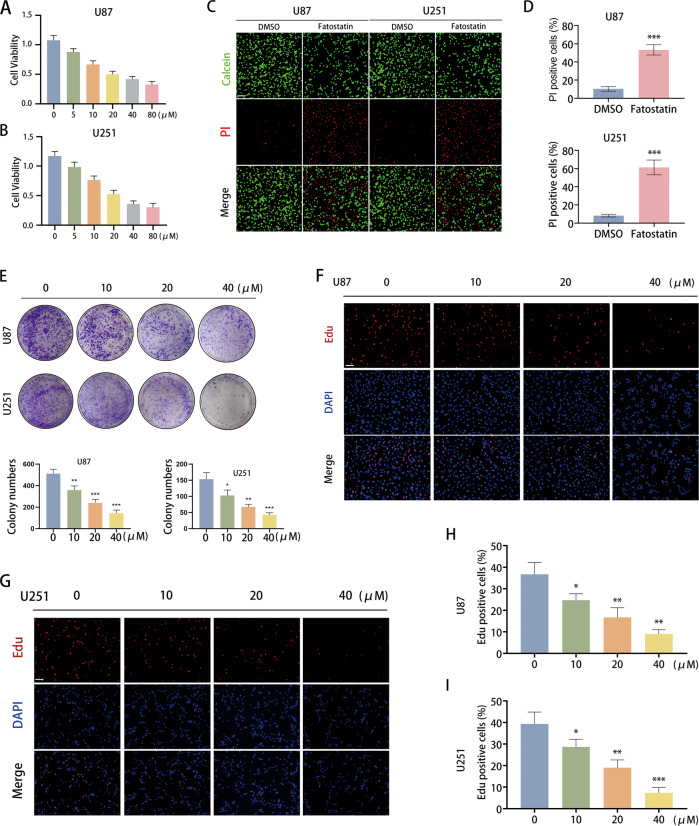
Fatostatin induces cell death and inhibits cell proliferation in GBM cells. **A**, **B** CCK-8 assays were used to measure the cell viability (absorbance value at 450 nm) of U87 and U251 cells after treatment with fatostatin. The IC50 of U87 cells was 21.38 μM, and the IC50 of U251 cells was 19.44 μM. **C**, **D** Live/Dead cell double staining assays were conducted in U87 and U251 cells. The percentage of PI-positive cells was calculated. Fatostatin was used at 20 μM. Scale bars: 50 μm. **E** Colony-formation assays were conducted in U87 and U251 cells. The colony numbers were counted by ImageJ. **F**–**I** EdU-DNA synthesis assays were used to evaluate cell proliferation. Representative images are shown in (**F**, **G**), scale bars: 50 μm. Quantification data show the percentage of EdU-positive U87 and U251 cells. *P < 0.05, **P < 0.01, ***P < 0.001.

Through the CCK-8 assay, we found that fatostatin could exert an inhibitory effect on cell viability in both U87 and U251 cells in a dose-dependent manner (Fig. 1A, B). Live/Dead cell double staining assays demonstrated that the number of dead cells stained with PI was significantly increased in the fatostatin treatment groups (Fig. 1C, D). The results of the colony-formation assay showed that fatostatin dramatically reduced the colony numbers of U87 and U251 cells in a dose-dependent manner (Fig. 1E). In addition, we conducted an EdU-DNA synthesis assay to assess the effect of fatostatin on cell proliferation, and the results showed that EdU-positive cells decreased significantly with increasing fatostatin dose (Fig. 1F–I). These results revealed that fatostatin could induce cell death and inhibit cell proliferation in GBM cell lines.

### Functional enrichment analysis of DEGs obtained from RNA-seq

**Fig. 2:**
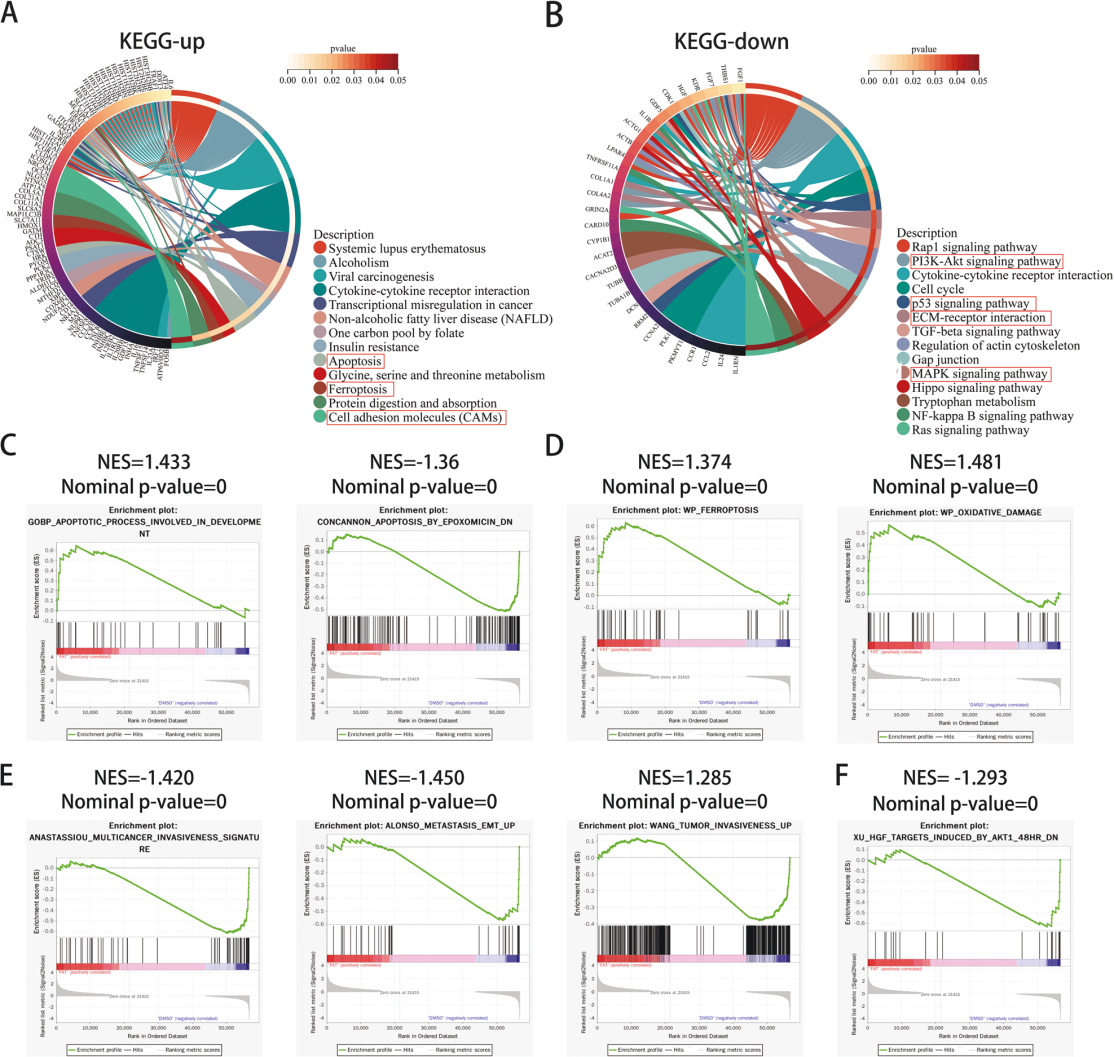
Functional enrichment analysis of DEGs obtained from RNA-seq. **A** KEGG enrichment analysis of upregulated DEGs. **B** KEGG enrichment analysis of downregulated DEGs. The outermost circle on the right represents the term in the lower right corner, and the inner circle on the right represents the *P* value of the corresponding pathway. **C**–**F** GSEA enrichment analysis between the DMSO group and the fatostatin group. NES normalized enrichment score.

To further explore the effect of fatostatin on GBM cells, we treated U87 cells with DMSO (as a control group) and fatostatin respectively (repeated three times) and performed transcriptome sequencing. Next, we performed differentially expressed gene analysis on the sequencing results, and the heatmap and volcano map of the results are shown in Supplementary Fig. 1. A total of 733 differentially expressed genes were obtained, of which 185 were expressed at low levels, and 548 were expressed at high levels in the fatostatin group. KEGG enrichment analysis showed that genes upregulated in the fatostatin group were involved in apoptosis, ferroptosis, and cell adhesion molecule pathways, while genes downregulated in the fatostatin group were mainly related to the PI3K-Akt signaling pathway, p53 signaling pathway, MAPK signaling pathway, ECM–receptor interaction and other pathways (Fig. 2A, B). Reactome enrichment analysis showed that these DEGs are related to oxidative stress-induced senescence, cellular responses to stress, the cell cycle, and ECM proteoglycans (Supplementary Fig. 1C, D). The results of GO enrichment analysis are also shown in Supplementary Fig. 1E, F, and these genes are mainly related to the cell cycle and immune response. In addition, GSEA was performed between upregulated and downregulated genes. We found that these genes were enriched in apoptosis, ferroptosis, oxidative damage, metastasis EMT, tumor invasiveness and the AKT pathway (Fig. 2C–F). To some extent, the above RNA-seq results provide directions for our subsequent experiments.

### Fatostatin attenuates the EMT process of GBM cells

**Fig. 3:**
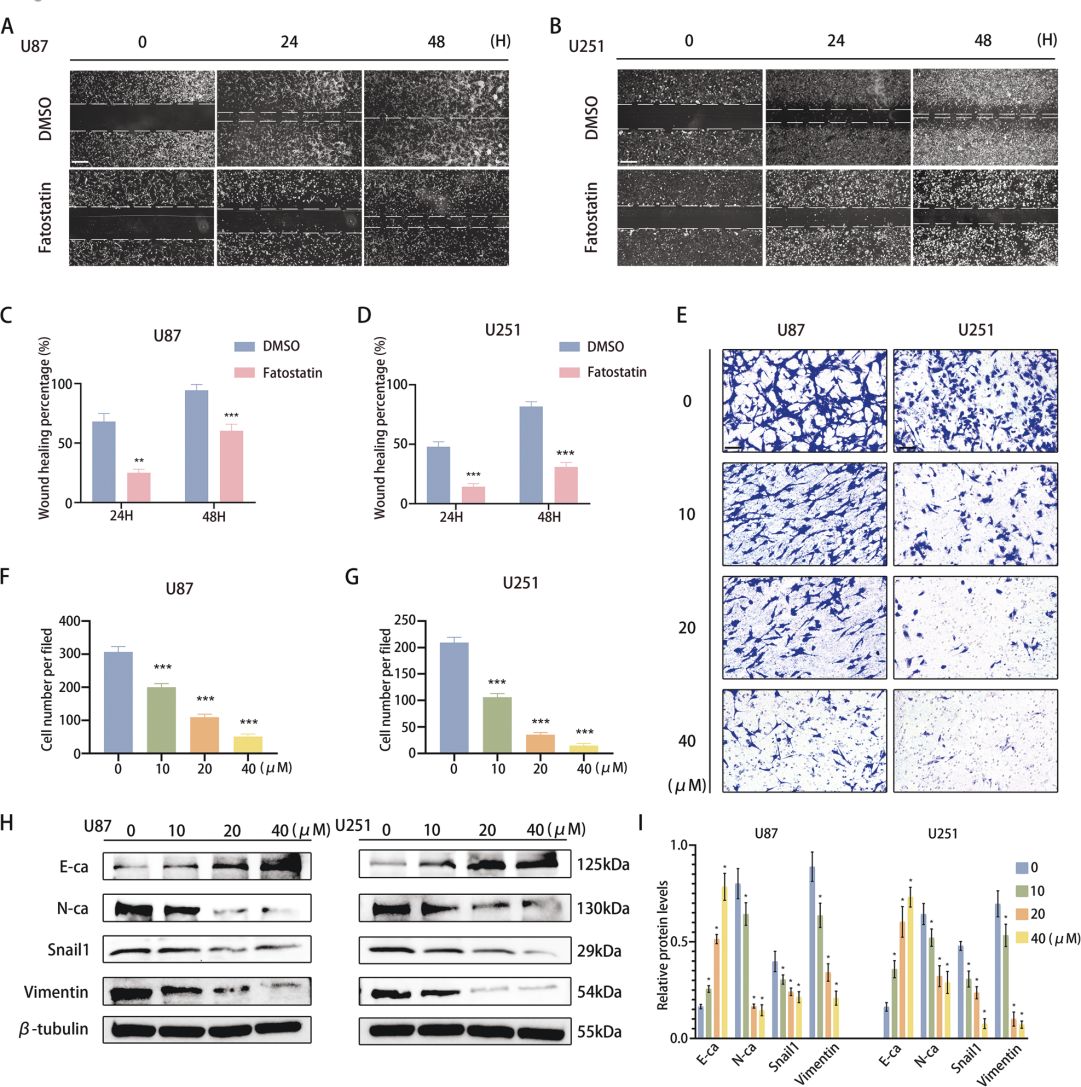
Fatostatin attenuates the EMT process of GBM cells. **A**–**D** Wound-healing assays were used to detect the migration of U87 and U251 cells after fatostatin treatment. Scale bars: 200 µm. **E**–**G** Transwell assays were used to detect the invasion of U87 and U251 cells after fatostatin treatment. Scale bars: 50 µm. **H**, **I** Western blot analysis was used to detect the expression of E-ca, N-ca, Snail1, and Vimentin in U87 and U251 cells after fatostatin treatment for 24 h. *P < 0.05, **P < 0.01, ***P < 0.001.

According to the above results, we explored the effect of fatostatin on the migration and invasion of GBM. Through a wound-healing assay, we found that compared with DMSO, fatostatin significantly attenuated the migration of U87 and U251 cells (Fig. 3A–D). Similarly, Transwell assays revealed that fatostatin decreased the invasion of GBM cell lines in a dose-dependent manner (Fig. 3E–G). To further explore the role of fatostatin in EMT, we performed Western blotting analysis of mesenchymal-related genes, including E-cadherin, N-cadherin, snail1, and vimentin, in GBM cells. The results showed that the expression of N-cadherin, snail1, and vimentin was decreased in a dose-dependent manner after treatment with fatostatin, while the expression of E-cadherin was upregulated (Fig. 3H, I). These results indicated that fatostatin might attenuate or even reverse the process of EMT in both U87 and U251 cell lines.

### Fatostatin induces ferroptosis mediated by GPX4 in GBM cells

**Fig. 4:**
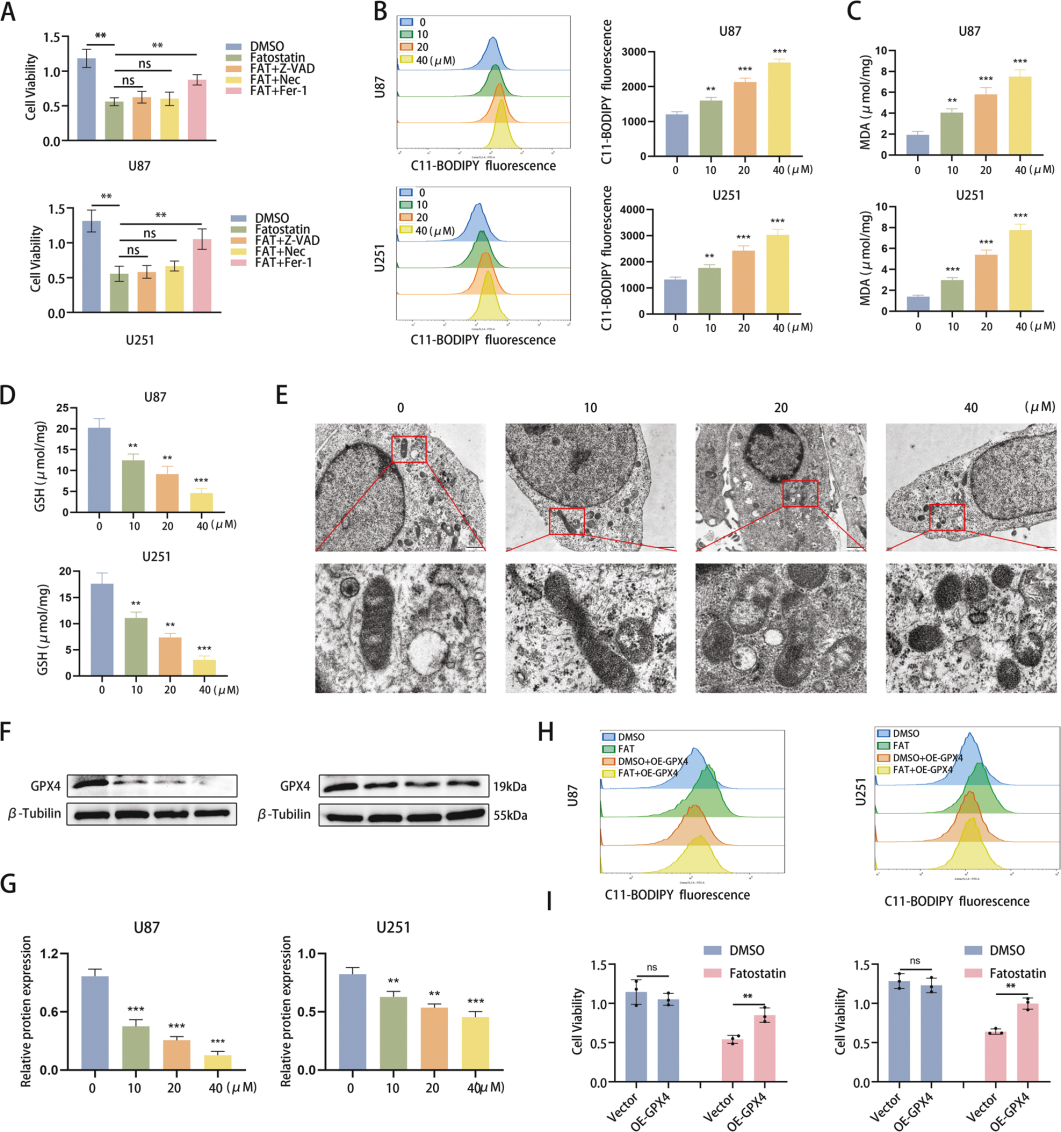
Fatostatin induces ferroptosis mediated by GPX4 in GBM cells. **A** According to the cell viability assay, Fer-1 (10 µM) reversed the decreased cell viability (absorbance value at 450 nm) of U87 and U251 cells induced by fatostatin, while Z-VAD (20 µM) and Nec (20 µM) did not. Z-VAD (Z-VAD-FMK), Fer-1 ferrostatin-1, Nec necrosulfonamide. **B** Flow cytometry was used to detect lipid ROS in U87 and U251 cells after fatostatin treatment. **C**, **D** MDA and GSH levels in U87 and U251 cells after fatostatin treatment were detected. **E** After DMSO and fatostatin treatment (20 µM), U87 cells were prepared for transmission electron microscopy observation. **F**, **G** The expression level of GPX4 in U87 and U251 cells after fatostatin treatment for 24 h. **H** Flow cytometry results showed that overexpression of GPX4 reversed the lipid ROS level in U87 and U251 cells. **I** CCK-8 assays showed that overexpression of GPX4 preserved cell viability (absorbance value at 450 nm) in U87 and U251 cells upon fatostatin treatment. *P < 0.05, **P < 0.01, ***P < 0.001; ns no significance.

Based on the enrichment analysis results, we further explored the manner in which fatostatin induces cell death. We used several cell death inhibitors (Z-VAD-FMK, a classic apoptosis inhibitor; necrosulfonamide, a necroptosis inhibitor; and ferrostatin-1, a ferroptosis inhibitor) to treat GBM cells and found that only the ferroptosis inhibitor reversed the effects of fatostatin (Fig. 4A). In addition, the colony-formation assay showed that ferrostatin-1 reversed the reduction in colony number induced by fatostatin (Supplementary Fig. 2A). Therefore, we next detected the changes in GSH and lipid oxidation (including lipid ROS and MDA) produced during ferroptosis. The results revealed that fatostatin significantly increased the lipid ROS level and MDA level in a dose-dependent manner (Fig. 4B, C). Likewise, ferrostatin-1 reversed the increased lipid ROS level induced by fatostatin (Supplementary Fig. 2B). GSH levels were obviously decreased after treatment with fatostatin (Fig. 4D). Next, TME was used to observe the morphological changes in the microstructure of U87 cells. In the fatostatin treatment groups, we observed typical ferroptotic characteristics, including shrunken mitochondria, shrinking or disappearing mitochondrial cristae, and increased mitochondrial membrane density (Fig. 4E). In addition, deferoxamine (DFO), a kind of iron chelator, could reverse the effect of fatostatin, which confirmed the iron dependence of fatostatin-induced death (Supplementary Fig. 2C).

Furthermore, we explored the mechanism by which fatostatin regulates ferroptosis in GBM cells by detecting the expression of ACSL4, SLC7A11, FTL, and GPX4, which are the core regulatory proteins of lipid oxidation and iron metabolism [21–23]. Our results revealed that only the expression of GPX4 was negatively altered with fatostatin treatment, while other molecules showed no significant change (Fig. 4F, G and Supplementary Fig. 2D). To further verify this finding, we designed a GPX4-overexpressing (OE-GPX4) plasmid with a flag tag to conduct rescue experiments, and the overexpression efficacy was ensured by western blotting (Supplementary Fig. 2E). Flow cytometry results showed that overexpression of GPX4 prevented the fatostatin-induced lipid ROS increase (Fig. 4H and Supplementary Fig. 2F). CCK-8 assays also showed that overexpressing GPX4 preserved cell viability in U87 and U251 cells upon fatostatin treatment (Fig. 4I). The above results suggested that fatostatin-induced ferroptosis in U87 and U251 cells is mainly mediated by GPX4.

### Fatostatin decreases GPX4 protein synthesis through the AKT/mTORC1/4EBP1 axis

**Fig. 5:**
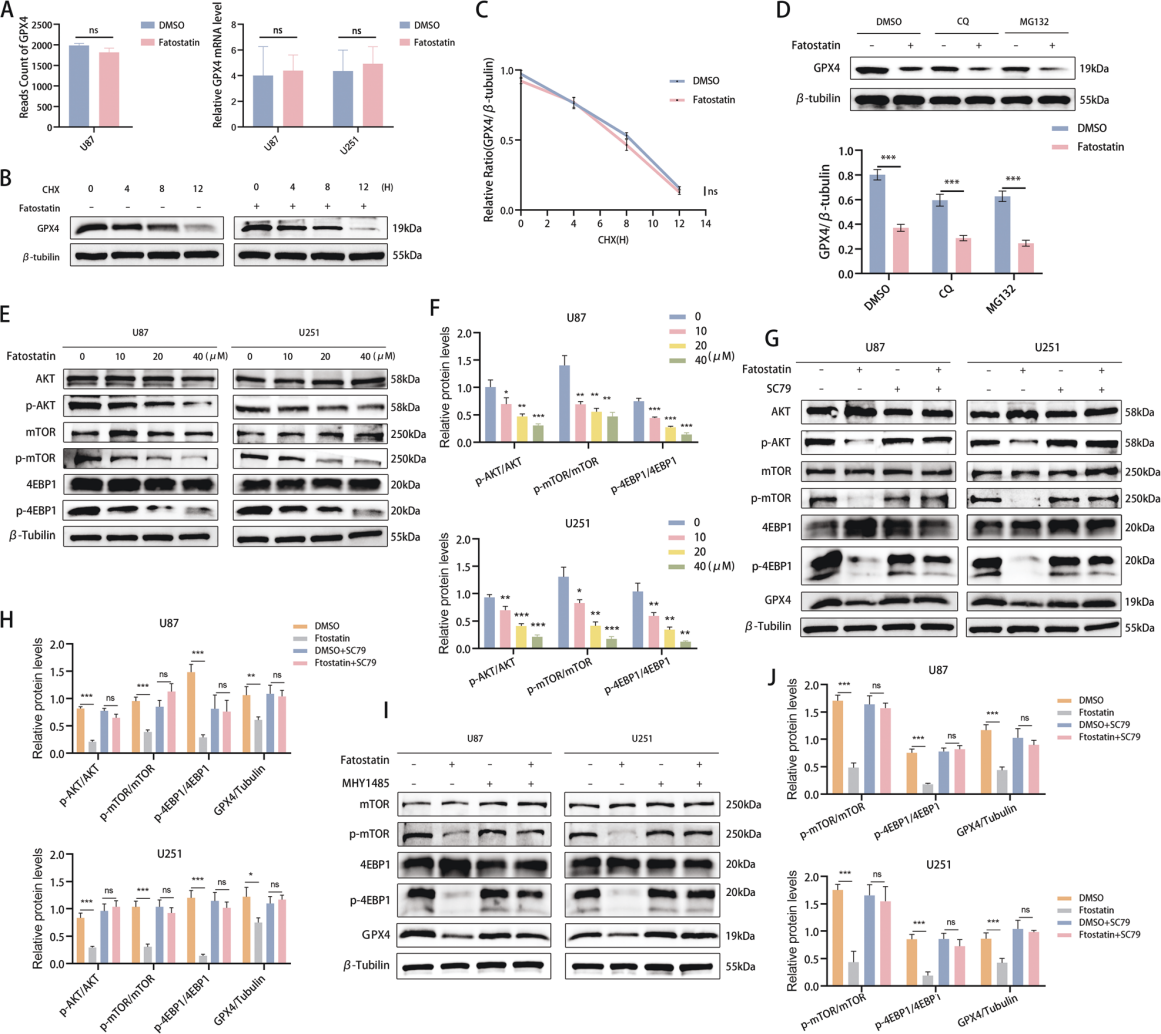
Fatostatin decreases GPX4 protein synthesis through the AKT/mTORC1/4EBP1 axis. **A** RNA-seq and RT‒PCR results showed that the mRNA level of GPX4 was not significantly different between the DMSO and fatostatin groups. **B**, **C** Western blotting was used to analyze the GPX4 protein levels after the cells were treated with or without fatostatin (20 μM) for 24 h and CHX (50 μM) for different durations (0, 4, 8, 12 h). **D** Western blot analysis of the GPX4 protein levels in the U87 cells treated with DMSO, CQ (lysosomal inhibitor, 10 μM), or MG132 (proteasomal inhibitor, 10 μM) with or without fatostatin (20 μM) treatment for 24 h. Lower panels show the statistical analysis of the data, *n* = 3. **E**, **F** The protein expression levels of AKT, p-AKT, mTOR, p-mTOR, 4EBP1, and p-4EBP1 in U87 and U251 cells after fatostatin treatment for 24 h. **G**, **H** SC79 (AKT activator, 10 μM) reversed the relative protein expression levels of p-AKT, p-mTOR, p-4EBP1, and GPX4 in the U87 and U251 cells treated with fatostatin (20 μM) for 24 h. **I**, **J** MHY1485 (mTOR activator, 10 μM) reversed the changes in relative protein expression levels of p-mTOR, p-4EBP1, and GPX4 in the U87 and U251 cells treated with fatostatin (20 μM) for 24 h. *P < 0.05, **P < 0.01, ***P < 0.001; ns no significance.

We then explored the upstream modulators of GPX4 in fatostatin-induced ferroptosis. Our RNA-seq results showed no significant changes in the mRNA levels of GPX4 in the fatostatin treatment group, and this result was also verified by our RT‒PCR assays (Fig. 5A). Considering that the GPX4 protein levels were decreased while its mRNA levels did not have corresponding changes, we speculated that GPX4 protein synthesis decreased or GPX4 protein degradation increased (or both). Therefore, we used cyclohexane (CHX), a protein synthesis inhibitor, to detect the half-life of the GPX4 protein in U87 cells by the CHX chase assay. Our results showed that there was no significant difference in the half-life of the GPX4 protein between the DMSO and fatostatin groups (Fig. 5B, C). Moreover, the GPX4 protein levels were not restored by treatment with the lysosome inhibitor chloroquine (CQ) or the proteasome inhibitor MG132 under fatostatin treatment (Fig. 5D). Therefore, we concluded that fatostatin decreases GPX4 protein levels by inhibiting GPX4 protein synthesis.

The mTORC1 pathway plays a crucial role in protein synthesis, and Zhang et al. found that the activation of mTORC1/4EBP could promote GPX4 protein synthesis in cancer cells [24]. Combined with our enrichment analysis results (Fig. 2B, J and Supplementary Fig. 3), the data showed that the AKT/mTOR signaling pathway may be affected after fatostatin treatment. Thus, we detected the expression levels of AKT, mTOR, 4EBP1, and their corresponding phosphorylated proteins by Western blotting. The results showed that the relative expression levels of p-AKT, p-mTOR, and p-4EBP1 decreased in a dose-dependent manner after fatostatin treatment in both U87 and U251 cells (Fig. 5E, F). Next, we performed rescue experiments using the AKT agonist SC79, and we observed that SC79 reversed the fatostatin-induced decrease in p-AKT, p-mTOR, p-4EBP1, and GPX4 protein expression in both GBM cell lines (Fig. 5G, H). Similar results were observed when we used the mTOR agonist MHY1485 to perform rescue experiments (Fig. 5I, J). In summary, we showed that the effect of fatostatin on GPX4 protein synthesis is mediated by the AKT/mTORC1/4EBP1 axis.

### Fatostatin inhibits proliferation and EMT and induces ferroptosis by suppressing the AKT/mTORC1 pathway

**Fig. 6:**
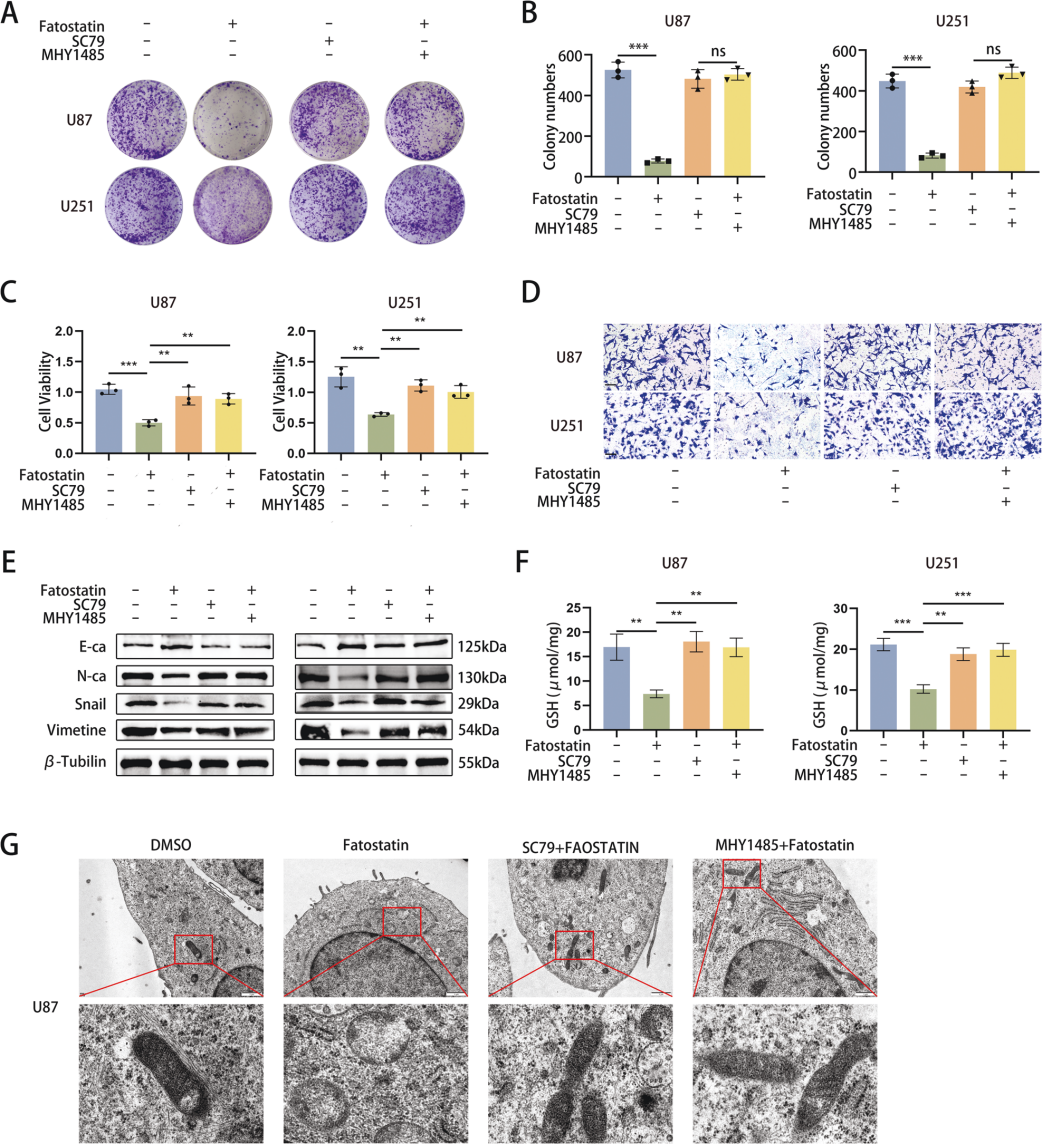
Fatostatin inhibits proliferation and EMT and induces ferroptosis by suppressing the AKT/mTORC1 pathway. **A**, **B** The changes in colony numbers in U87 and U251 cells were reversed by SC79 or MHY1485. **C** CCK-8 assay results showed that the changes in U87 and U251 cells were reversed by SC79 or MHY1485. **D** Transwell assays revealed that the changes in the invasion of U87 and U251 cells were reversed by SC79 or MHY1485. **E** The changes in expression of E-ca, N-ca, Snail1, and Vimentin in U87 and U251 cells treated with fatostatin (20 μM) for 24 h were reversed by SC79 or MHY1485. **F** The changes in the GSH levels in U87 and U251 cells were reversed by SC79 or MHY1485. **G** After DMSO, fatostatin, fatostatin+SC79, or fatostatin+MHY1485 treatment, U87 cells were prepared for transmission electron microscopy observation. *P < 0.05, **P < 0.01, ***P < 0.001; ns no significance.

Subsequently, we further investigated the effects of the AKT/mTORC1 pathway on cell proliferation, EMT, and ferroptosis. Colony-formation assays revealed that both SC79 and MHY1485 could reverse the decrease in colony numbers induced by fatostatin treatment (Fig. 6A, B). The CCK-8 assay demonstrated that SC79 and MHY1485 could restore cell viability after fatostatin treatment in U87 and U251 cells (Fig. 6C). Upon activation of the AKT/mTORC1 pathway, the fatostatin-induced decrease in the invasive capacity of GBM cells as well as the expression of EMT-related proteins (including E-cadherin, N-cadherin, snail1, and vimentin) was also reversed (Fig. 6D, E). We examined the changes in GSH levels upon the addition of SC79 and MHY1485 and found that the fatostatin-induced decrease in GSH levels was also reversed (Fig. 6F). We observed the microstructure of the cells by TEM and found that the mitochondrial morphology no longer had the typical characteristics of ferroptosis when SC79 or MHY1485 was added (Fig. 6G). The above results suggest that fatostatin regulates cell proliferation, EMT and ferroptosis through the AKT/mTORC1 pathway.

### Engineered fatostatin NPs enhance the antitumor effects of fatostatin through targeted delivery to GBM cells

**Fig. 7:**
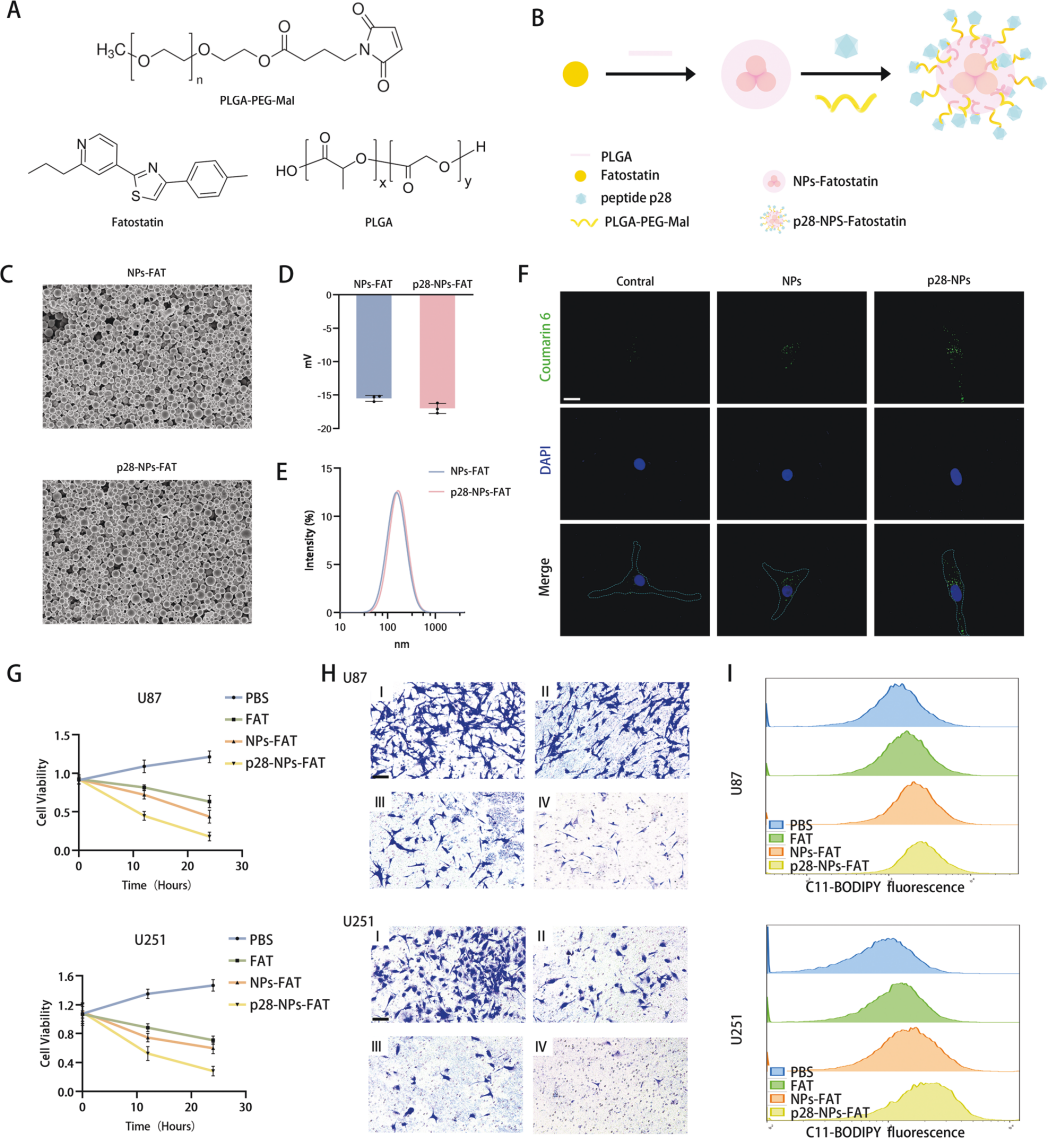
Engineered fatostatin NPs enhance the antitumor effects of fatostatin through targeted delivery to GBM cells. **A** The chemical formula of fatostatin, PLGA, and PLGA-PEG-MAL. **B** Schematic diagram of nanoparticle synthesis. **C** SEM showed the morphology of NPs-FAT (loaded with fatostatin) and p28-NPs-FAT. **D** The ζ-potential of NPs-FAT and p28-NPs-FAT. **E** The mean hydration diameter of NPs-FAT and p28-NPs-FAT. **F** Cellular uptake assay using free C6, NPs encapsulated C6, and p28-NPs encapsulated C6. **G** CCK-8 assays showed the cell viability (absorbance value at 450 nm) of U87 and U251 cells after treatment with PBS, fatostatin (FAT), NPs-FAT, or p28-NPs-FAT. **H** The invasion ability of U87 and U251 cells after treatment with PBS (I), FAT(II), NPs-FAT(III), or p28-NPs-FAT (IV). **I** The ROS levels of U87 and U251 cells after treatment with PBS, FAT, NPs-FAT, or p28-NPs-FAT.

To better deliver the drug to the tumor, we designed and synthesized p28-functionalized PLGA nanoparticles loaded with fatostatin (p28-NPs-FAT). Figure 7A, B shows the chemical formulas of fatostatin, PLGA, and PLGA-PEG-MAL and a schematic diagram of nanoparticle synthesis. SEM results showed that the morphology of PLGA NPs-FAT (loaded with fatostatin) was not changed after surface modification with the p28 peptide (Fig. 7C). The DLS results showed that their ζ-potentials were −15.5 ± 0.44 mV and −17 ± 0.81 mV (Fig. 7D), and their mean hydration diameter was approximately 140 nm (Fig. 7E). There was also no significant difference in hydration diameter and ζ-potential between the two NPs. To investigate the ability of GBM cells to internalize NPs and p28-NPs-FAT, we performed a cellular uptake assay using synthetic NP-encapsulated C6, a fluorescent dye that allows noninvasive imaging. The results showed that the control group had very few internalized fluorescent particles, while fluorescent NPs diffused evenly throughout the cytoplasm in the NPs groups and p28-NPs group. In addition, compared to the NPs group, the p28-NPs group had significantly more internalized fluorescent particles (Fig. 7F). Next, we continued to investigate the effects of NPs-FAT and p28-NPs-FAT on proliferation, EMT, and ferroptosis in GBM cell lines. We first demonstrated that free NPs and p28-NPs had no effect on the cell viability of U87 and U251 cells (Supplementary Fig. 4A, B). Then, we found that p28-NPs-FAT exerted the strongest effect in inhibiting cell viability and cell invasion and promoting the production of lipid ROS during ferroptosis at the equivalent dose of fatostatin (Fig. 7G–I). Unsurprisingly, ferrostatin-1 also reversed the decreased cell viability in U87 and U251 cells induced by NPs-FAT and p28-NPs-FAT (Supplementary Fig. 4C, D). These results suggest that fatostatin NPs enhanced the cellular uptake of fatostatin and better inhibited GBM cells in vitro.

### Fatostatin NPs target GBM and inhibit GBM growth in an intracranial xenograft model

**Fig. 8:**
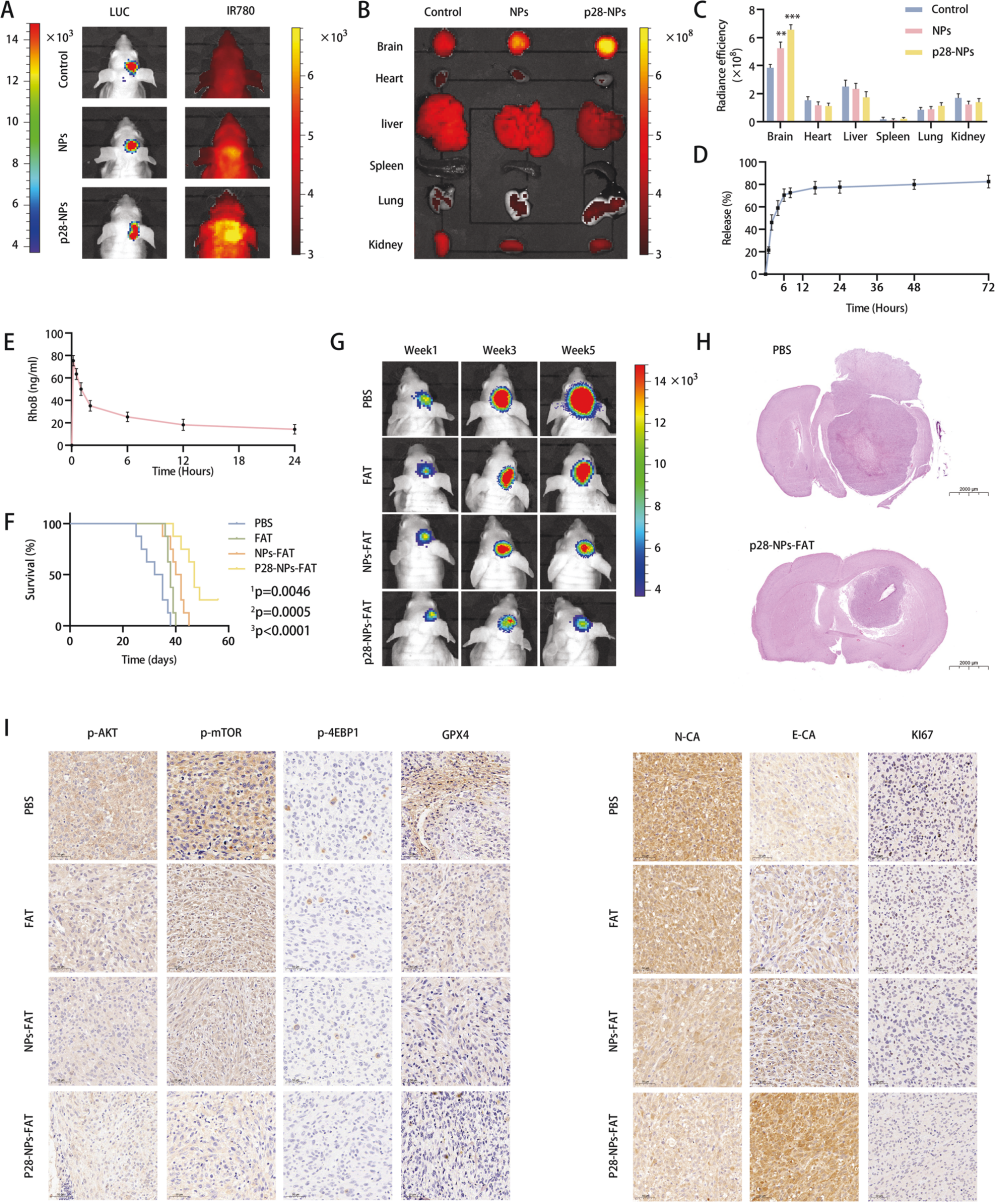
Fatostatin NPs target GBM and inhibit GBM growth in an intracranial xenograft model. **A** Representative IVIS imaging of the tumor-bearing mice treated with free IR780 (control), NPs loaded with IR780, and p28-NPs loaded with IR780, respectively. The left image is luciferase fluorescence indicating the tumor size, and the right image is the IR780 signal indicating the accumulation of IR780 particles. **B** IVIS imaging of the isolated organs showed the IR780 signal in the brain, heart, liver, spleen, lung, and kidney of mice receiving the corresponding treatments. **C** The semiquantification of the NPs-IR780 signal from the isolated organs. **D** The controlled release of p28-NPs-FAT in PBS. **E** The plasma concentration of RhoB was detected at predetermined time intervals after intravenous injection of p28-NPs loaded with RhoB. **F** Kaplan‒Meier curves showing the survival of the untreated or treated mice in the experimental groups. (^1^p: comparing the PBS with fatostatin (FAT) treatment group; ^2^p: comparing the PBS with NPs-FAT treatment group; ^3^p: comparing the PBS with p28-NPs-FAT treatment group.) **G** Representative bioluminescence images from IVIS imaging showed the tumor luciferase signal in the mice of different treatment groups at 1, 3, and 5 weeks. **H** Representative images of H&E staining of brain sections of the different groups. **I** Representative IHC images showing the expression levels of p-AKT, p-mTOR, p-4EBP1, GPX4, E-ca, and N-ca in brain sections.

To evaluate the delivery efficiency of NPs, we treated three groups of tumor-bearing mice with free IR780 (control), NPs loaded with IR780, and p28-NPs loaded with IR780, respectively. IVIS imaging showed that the mice in the control group had essentially no IR780 particles intracranially, while p28-modified NPs significantly enhanced the accumulation of IR780 particles (Fig. 8A). Similarly, IVIS imaging showed no significant difference in the fluorescence intensity of isolated organs except for the brain, and the liver and kidney had the highest level of fluorescence among the peripheral organs (Fig. 8B, C). These results suggest that p28-NPs could better cross the blood‒brain barrier and target intracranial GBM. Next, we performed in vitro release experiments of p28-NPs-FAT and found that more than 50% of fatostatin was released within the first 4 h, followed by sustained release over 72 h (Fig. 8D). Moreover, the pharmacokinetics of p28-NPs in mice were detected, and the results showed that the half-life of p28-NPs was ~12.54 h (Fig. 8E).

We assessed fatostatin and fatostatin NPs for GBM treatment in an intracranial xenograft model. Through IVIS imaging, we demonstrated that free NPs and p28-NPs had no effect on tumor growth in an intracranial xenograft model (Supplementary Fig. 4E). Next, we randomly divided the GBM-bearing mice into four groups and administered PBS, free fatostatin, NPs-FAT, and p28-NPs-FAT treatments, respectively. The survival analysis results showed that free fatostatin, NPs-FAT, and p28-NPs-FAT all prolonged the survival time of the mice, and the p28-NPs-FAT group had the longest survival time (Fig. 8F). IVIS imaging at 1, 3, and 5 weeks showed that free fatostatin, NPs-FAT, and p28-NPs-FAT all inhibited the growth of intracranial tumors in the mice, and p28-NPs-FAT had the strongest inhibitory effect on tumors (Fig. 8G). Similarly, HE staining revealed that compared to the mice in the PBS group, the mice in the p28-NPs-FAT treatment group had significantly smaller tumor volumes (Fig. 8H). We also examined the expression levels of p-AKT, p-mTOR, p-4EBP1, GPX4, E-CA, and N-CA in xenograft tumors by IHC, and the results were consistent with the findings in the in vitro experiments (Fig. 8I). The above results suggested that fatostatin could inhibit GBM growth to some extent in the intracranial xenograft model, while p28-NPs loaded with fatostatin could target GBM and better inhibit GBM growth.

## Discussion

Our results revealed that fatostatin treatment triggered GPX4-mediated ferroptotic cell death by repressing the AKT/mTORC1/GPX4 signaling pathway in GBM. Moreover, fatostatin-induced inhibition of cell proliferation and EMT was attributed to the AKT/mTORC1 signaling pathway. To overcome the hindrance of the BBB, we designed p28-functionalized PLGA nanoparticles loaded with fatostatin and demonstrated that they could significantly increase the dose of the drug reaching intracranial tumors and significantly inhibit tumor growth.

In other cancers, the antitumor activities of fatostatin have been previously linked to apoptosis caused by endoplasmic reticulum stress, cell cycle arrest, and inhibition of cancer cell proliferation, invasion, and migration [12, 25, 26]. In addition, most studies have focused on the inhibitory effect of fatostatin on SREBPs [11, 27, 28]. However, in GBM, we found for the first time that it induced ferroptosis but not apoptosis. The inactivation of GPX4 has been shown to be the predominant mechanism of ferroptosis because GPX4 is the only glutathione peroxidase used for liposome peroxidase reduction in cells [29]. Our results revealed that fatostatin significantly inhibited GPX4 synthesis in GBM cells in a dose-dependent manner. Interestingly, although we did not detect changes in SLC7A11, GSH changes were evident after fatostatin treatment. Therefore, we speculate that fatostatin may affect GSH levels by other mechanisms, such as affecting the rate-limiting enzyme of GSH synthesis. Similar to our results, Cheng et al. found that the activation of PI3K-AKT-mTOR signaling could enhance the expression of GPX4, thereby inhibiting ferroptosis in rheumatoid arthritis. Another report showed that cystine promotes GPX4 synthesis through activation of the mTORC1-4EBP signaling axis in a variety of tumor cell lines [24]. We reversed the changes in lipid ROS levels and cell viability by using a GPX4 overexpression plasmid, thereby identifying fatostatin-induced GPX4-mediated ferroptosis. After excluding factors of protein degradation, we determined that reduced GPX4 expression was mainly attributed to the inhibition of the AKT/mTORC1/GPX4 signaling pathway. In contrast to our study, Yi J et al. found that the activation of PI3K-AKT-mTOR signaling could suppress ferroptosis via SREBP/SCD1-mediated lipogenesis [30]. Although such findings have not been reported in GBM, combined with our results, these data indicate that the AKT-mTOR pathway plays multiple roles in ferroptosis in GBM, which should be further investigated. These findings in GBM are exciting, and we conducted animal studies to verify our findings in vivo. The IHC results of mouse intracranial tumors showed that the expression of p-AKT, p-mTOR, and GPX4 was significantly downregulated in the fatostatin (loaded in p28-NPs) treatment group compared to the control group. These findings extended the antitumor role of fatostatin and might provide a new perspective for the exploration of ferroptosis in GBM in the future.

Excessive cell proliferation is one of the hallmarks of tumors [30]. The dysregulation of AKT/mTOR has been reported to play a critical role in the proliferation of GBM cells, and several inhibitors of this pathway have been developed over the past few decades but have shown limited effects [31]. Studies have shown that fatostatin could inhibit migration, invasion, and the EMT process in esophageal carcinoma [27] and endometrial carcinoma [28]. Moreover, the AKT/mTOR pathway is involved in the EMT process [32] and is associated with the invasion and migration of GBM [33]. Consistent with their results, our findings revealed that fatostatin inhibited GBM cell proliferation and EMT, and AKT and mTOR agonists reversed fatostatin-induced cell proliferation and EMT. In the future, further exploration of the mechanism by which fatostatin inhibits proliferation and EMT may provide a new direction for the treatment of GBM.

For a long time, researchers have been searching for and developing new drugs to treat GBM, but most of them have unsatisfactory therapeutic effects, and one of the major reasons is the poor penetration of the drugs into the brain [31]. With the development of nanotechnology, various nanosized drug delivery systems have emerged as promising strategies for intracranial drug delivery. PLGA is one of the most promising drug delivery vehicles but has many limitations (including negative charge, hydrophobic structure, and nontargeting of the BBB), which adversely impact blood circulation time and the extent of NP uptake by target cells, and therefore does not show a satisfactory performance without modification [14]. PLGA can be easily chemically modified with various molecules, including peptides, aptamers, antibodies, dendrimers, and carbohydrates, which allows active targeting capabilities [19]. CPPs can efficiently enter multiple cell types without damaging cell membranes, making them a promising approach for drug-targeted delivery [34]. Peptide p28, a part of the bacterial protein azurin, is a kind of CPP that not only preferentially enters cancer cells over corresponding normal cells but also exhibits strong anticancer activity [35]. After entering the tumor, p28 can bind to p53 to inhibit its ubiquitination and proteasomal degradation, thus exerting an anticancer effect [36]. Recently, p28 was shown to preferentially localize to tumor sites in GBM orthotopic xenograft mouse models and enhance the antitumor effect of TMZ [37]. We innovatively combined PLGA with p28 to synthesize p28-functionalized PLGA nanoparticles loaded with fatostatin and applied them to the intracranial xenograft model. Unsurprisingly, in vitro experiments showed that p28-NPs-fatostatin enhanced the ability of fatostatin to induce ferroptosis and inhibit proliferation and EMT. Experiments in vivo showed that p28-NPs-fatostatin strongly enhanced the inhibitory effect of fatostatin on tumor growth and significantly prolonged the survival time of mice. In the future, we will continue to investigate whether there is synergy between p28 and fatostatin in GBM and the specific mechanisms underlying this synergy.

In conclusion, this study revealed a novel antitumor activity of fatostatin in GBM. Furthermore, our study highlighted the effect of AKT/mTORC1/GPX4 signaling on fatostatin-induced ferroptosis. We designed a previously unrecognized nanodelivery platform (p28-NPs-fatostatin) that can significantly improve the anti-GBM effect of fatostatin.

## Supplementary information

Supplementary Figure legends

Supplementary Figure1

Supplementary Figure2

Supplementary Figure3

Supplementary Figure4

AJE Editing Certificate

aj-checklist

Original Data File

## Data Availability

The original contributions presented in the study are included in the article/Supplementary Material; further inquiries can be directed to the corresponding author.
