# Supplementary material for: Fatostatin induces ferroptosis through inhibition of the AKT/mTORC1/GPX4 signaling pathway in glioblastoma
Source: Cell Death Dis. 2023 Mar 25;14(3):211. doi: 10.1038/s41419-023-05738-8 (PMC10039896; doi:10.1038/s41419-023-05738-8)
Supplement: Supplementary file 1 — Supplementary Figure legends [file 41419_2023_5738_MOESM1_ESM.docx]

**Supplementary Figure legends:**

**Supplementary Figure 1.** **(A, B)** Heatmap and volcano map of the differential expression results. **(C)** Reactome enrichment analysis of upregulated DEGs. **(D)** Reactome enrichment analysis of downregulated DEGs. **(E)** GO enrichment analysis of upregulated DEGs. **(F)** GO enrichment analysis of downregulated DEGs.

**Supplementary Figure 2.** **(A)** The changes in the colony numbers of U87 and U251 cells were reversed by ferrostatin-1. **(B)** Flow cytometry results showed that ferrostatin-1 reversed the change in the lipid ROS level in U87 and U251 cells. **(C)** CCK-8 assays showed that the change in cell viability (absorbance value at 450 nm) of U87 and U251 cells was reversed by deferoxamine (DFO) treatment (500 µM). **(D)** The expression levels of ACSL4, SLC7A11, and FTL in U87 and U251 cells after fatostatin treatment. **(E)** Western blot analysis confirmed the overexpression efficacy of the GPX4-overexpressing (OE-GPX4) plasmid with a flag tag. **(F)** Quantitative analysis of the lipid ROS levels in different groups. **P* < 0.05, ***P* <0.01, ****P* < 0.001; ns: no significance.

**Supplementary Figure 3.** GSEA enrichment analysis between the DMSO group and the fatostatin group. NES: normalized enrichment score.

**Supplementary Figure 4. (A)** Cell viability assay of the U87 cells treated with PBS, DMSO, NPs, and p28-NPs for 24 h. **(B)** Cell viability assay of the U251 cells treated with PBS, DMSO, NPs, and p28-NPs for 24 h. **(C)** The cell viability assay showed that ferrostatin-1 could reverse the decreased cell viability (absorbance value at 450 nm) in the U87 cells induced by NPs-FAT and p28-NPs-FAT. **(D)** The cell viability assay showed that ferrostatin-1 could reverse the decreased cell viability (absorbance value at 450 nm) in the U251 cells induced by NPs-FAT and p28-NPs-FAT. **(E)** Representative bioluminescence images from IVIS imaging showed the tumor luciferase signal in the mice of different treatment groups (PBS, DMSO, NPs, and p28-NPs) at 1, 3, and 5 weeks. **P* < 0.05, ***P* <0.01, ****P* < 0.001; ns: no significance.
