# Supplementary figures and images for: Fatostatin induces ferroptosis through inhibition of the AKT/mTORC1/GPX4 signaling pathway in glioblastoma
Source: Cell Death Dis. 2023 Mar 25;14(3):211. doi: 10.1038/s41419-023-05738-8 (PMC10039896; doi:10.1038/s41419-023-05738-8)

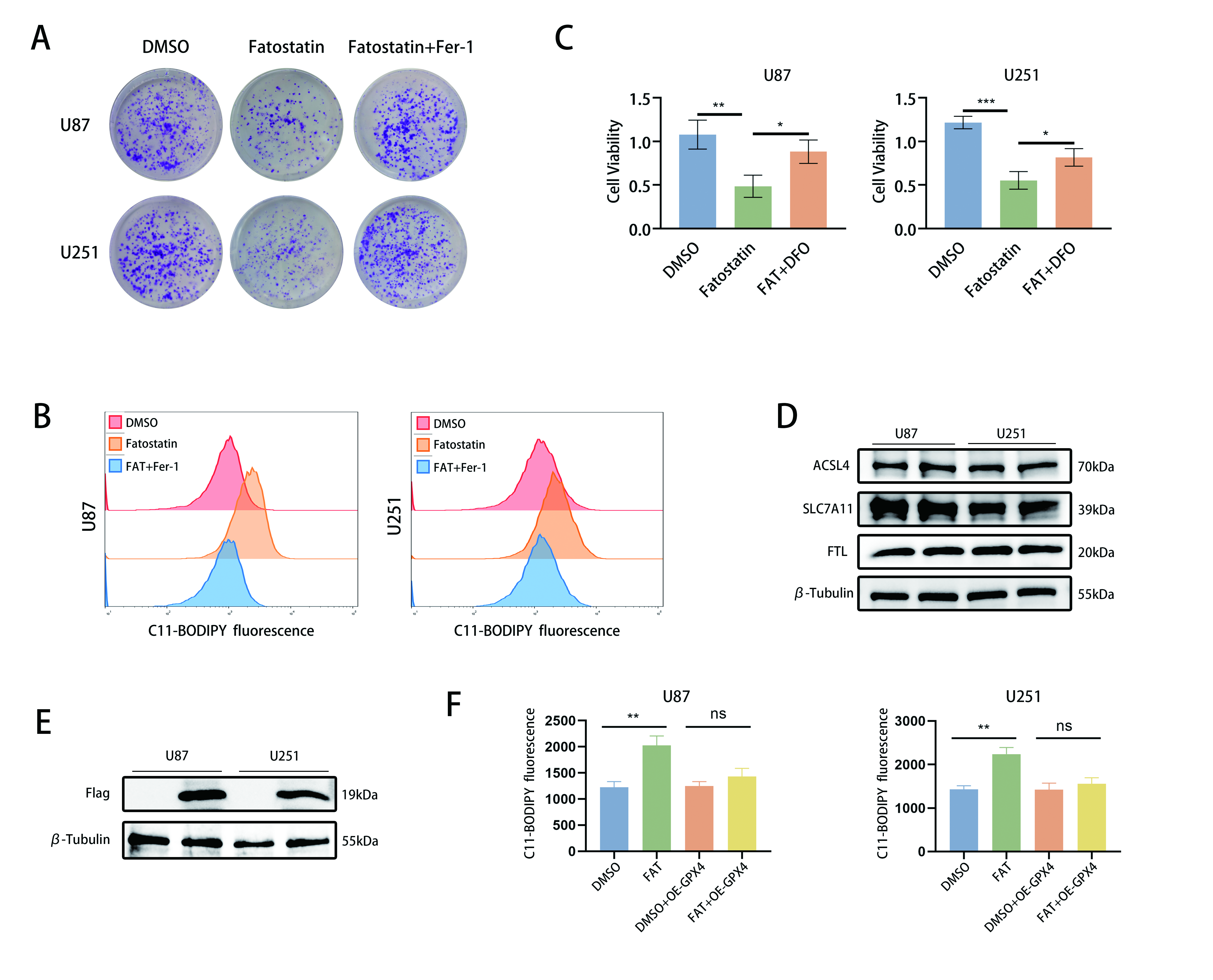

Supplement: Supplementary file 3 — Supplementary Figure2 [file 41419_2023_5738_MOESM3_ESM.tif]

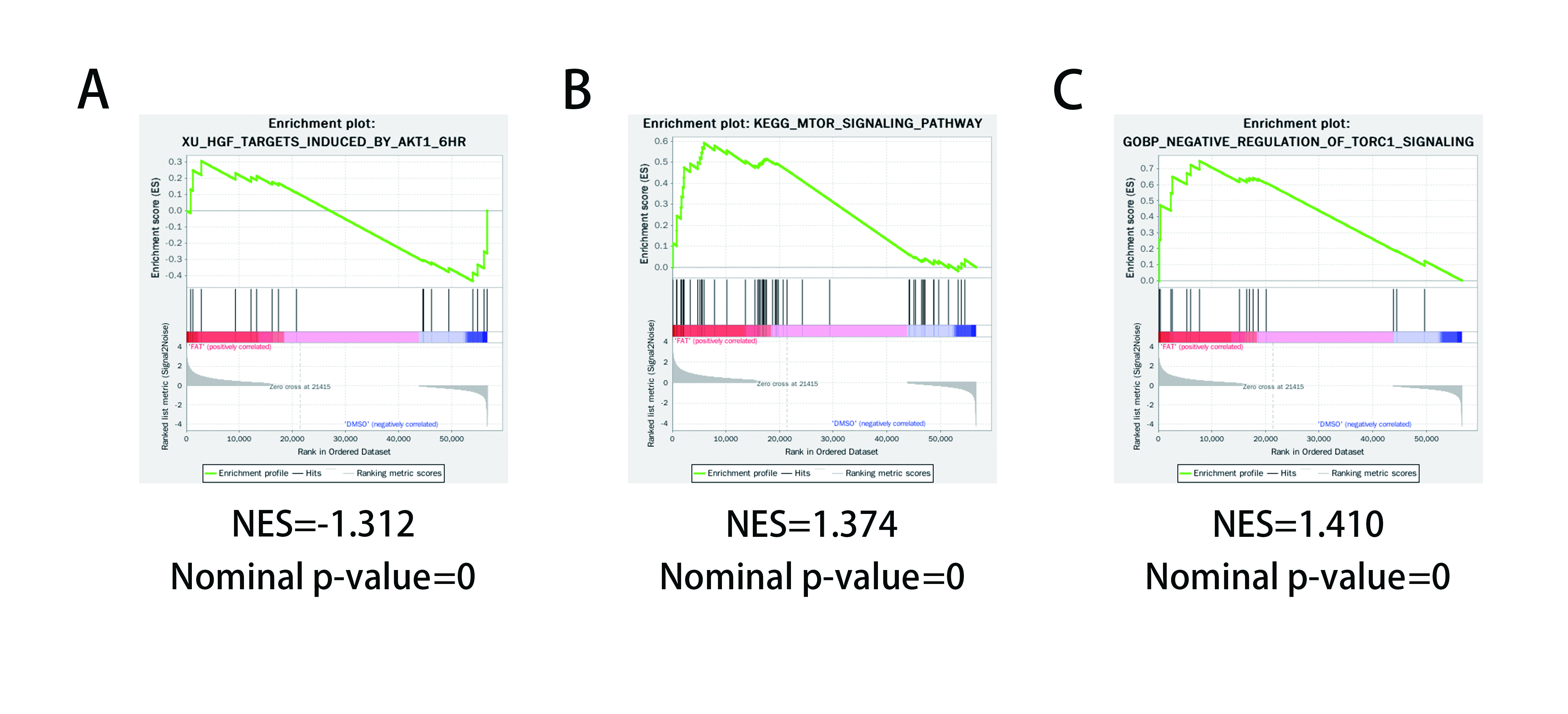

Supplement: Supplementary file 4 — Supplementary Figure3 [file 41419_2023_5738_MOESM4_ESM.tif]

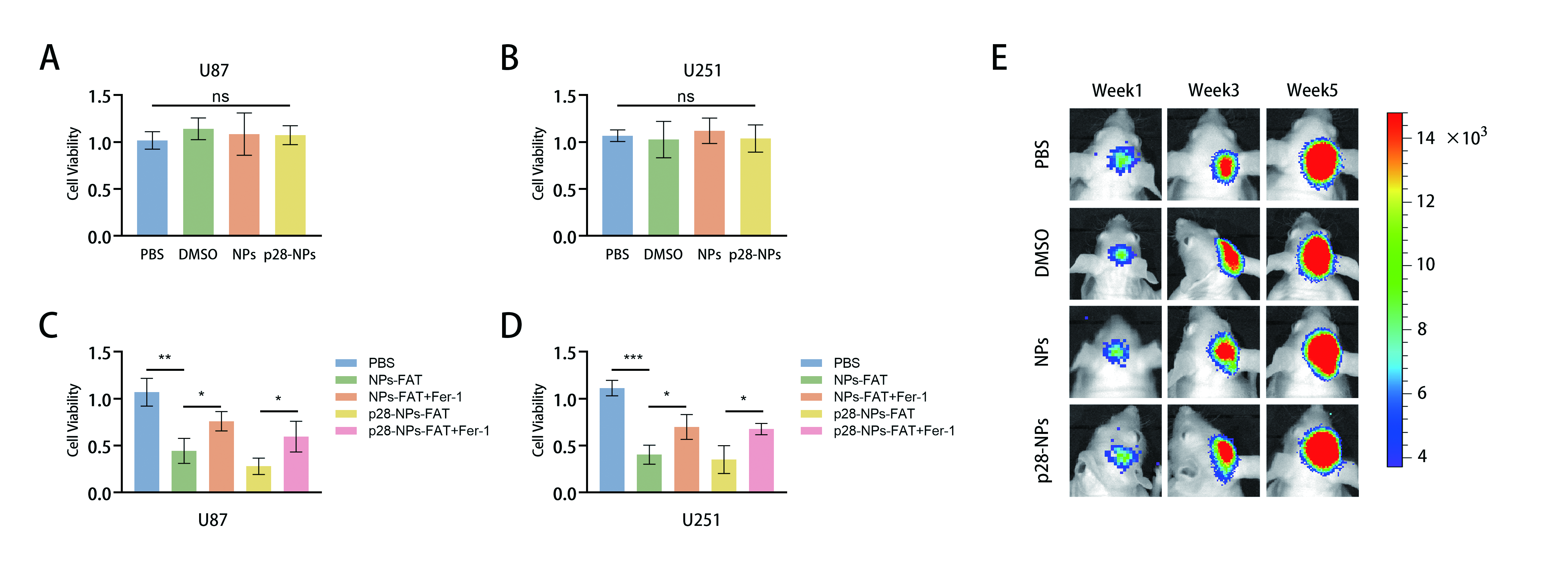

Supplement: Supplementary file 5 — Supplementary Figure4 [file 41419_2023_5738_MOESM5_ESM.tif]

**Figure 3**


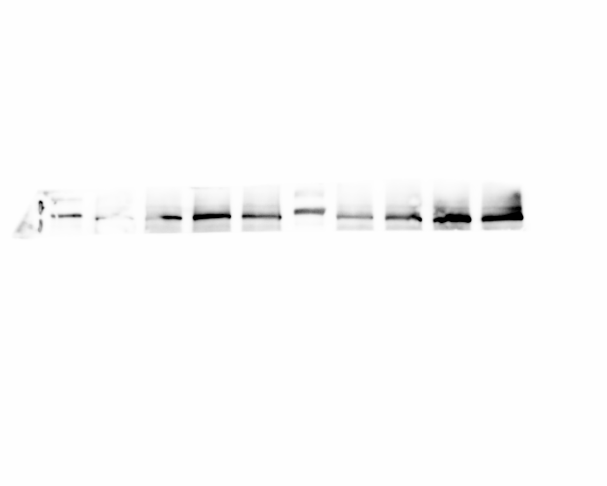

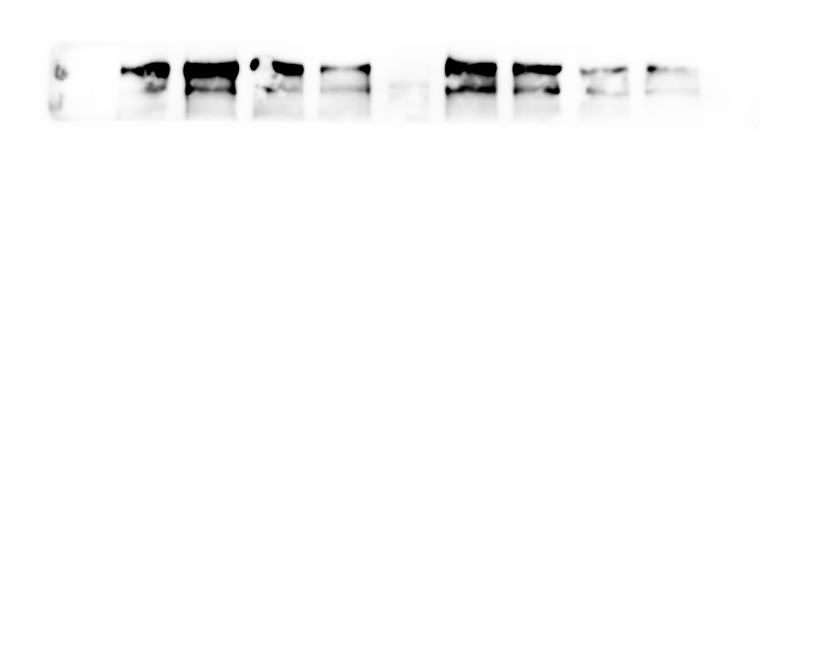

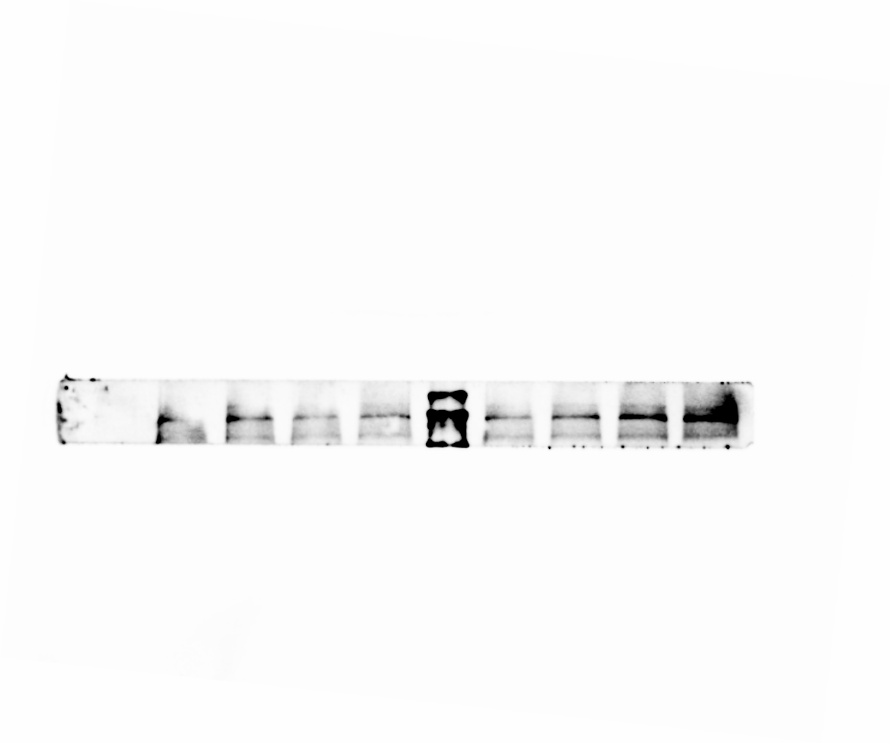


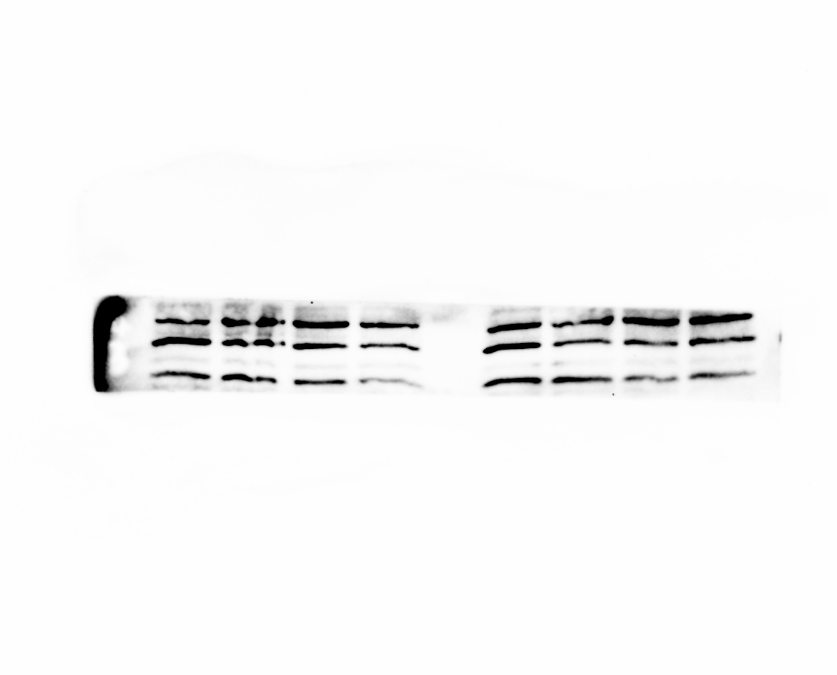

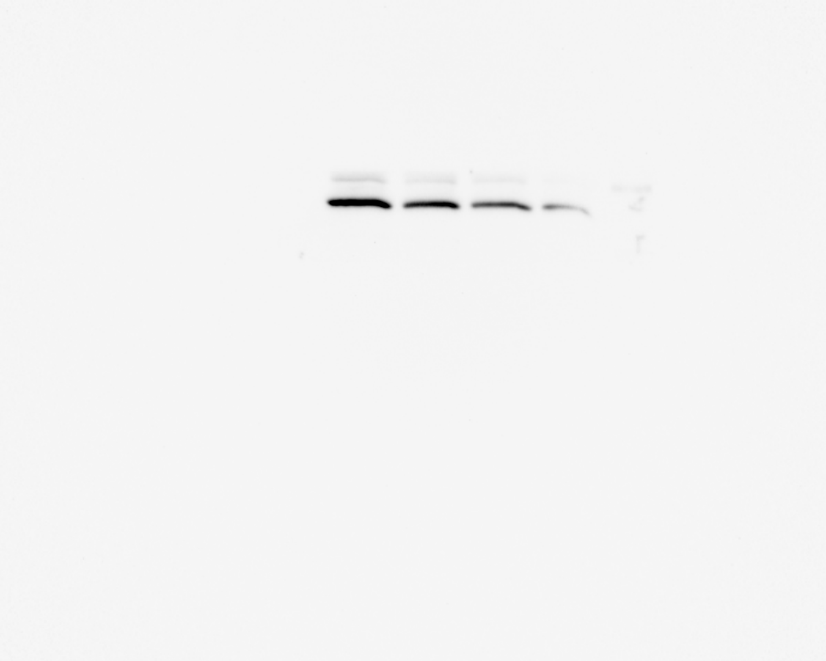


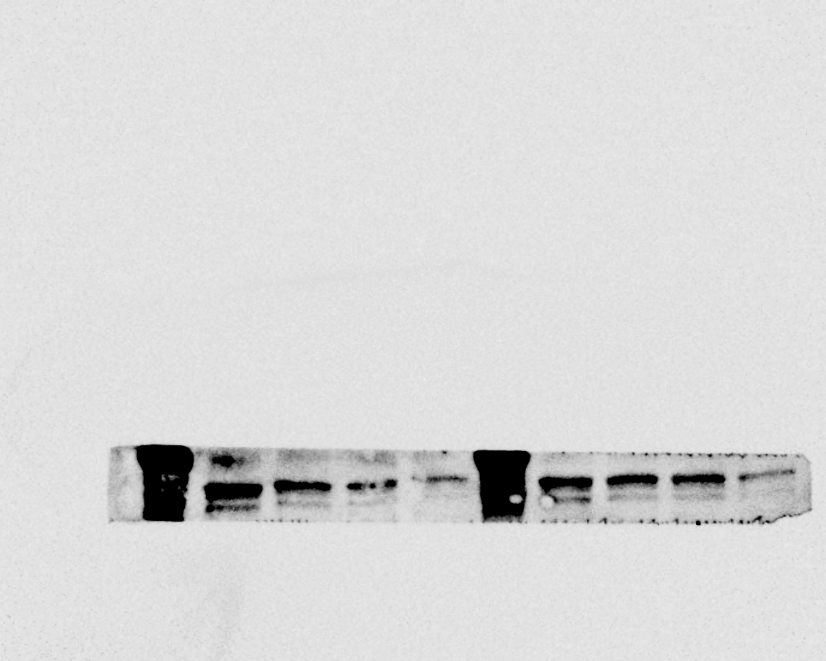

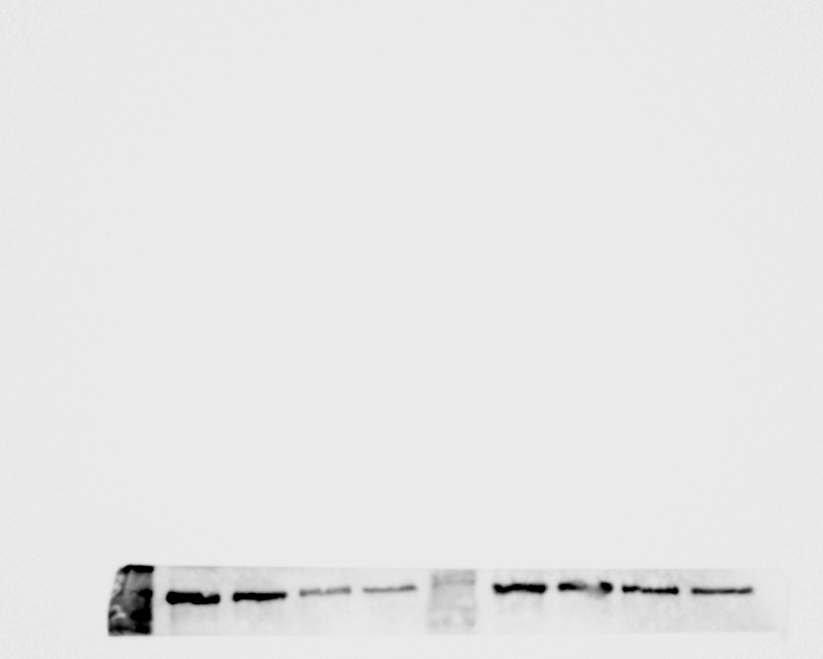


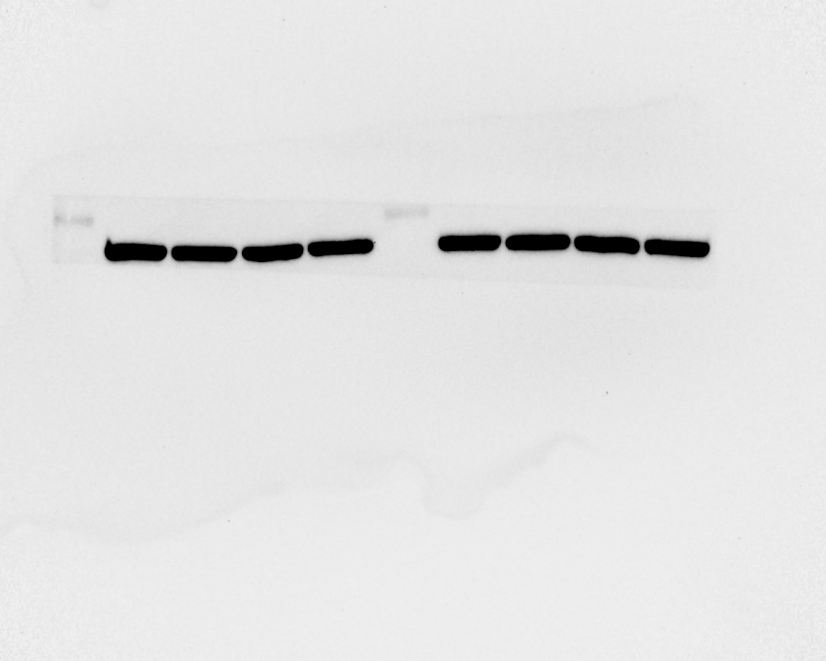


**Figure 4**


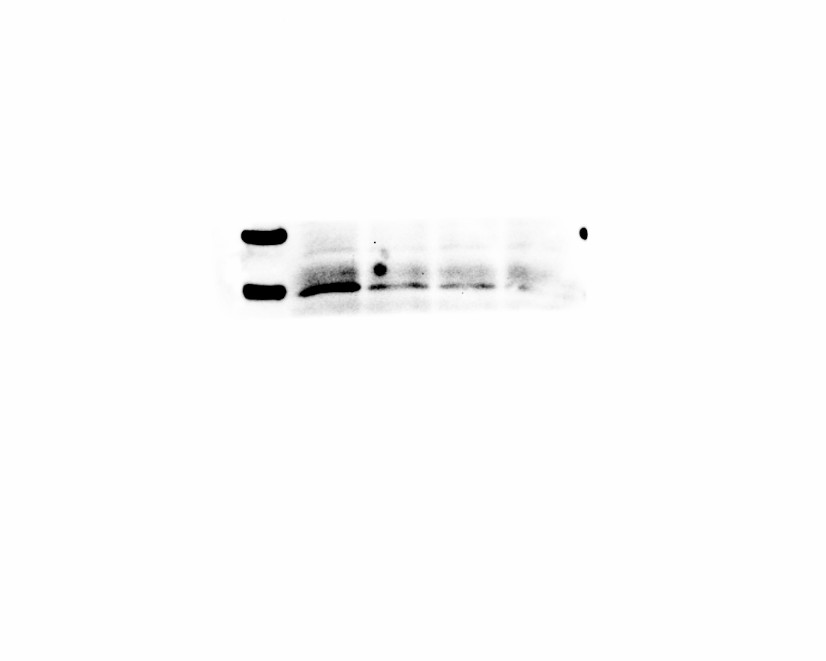

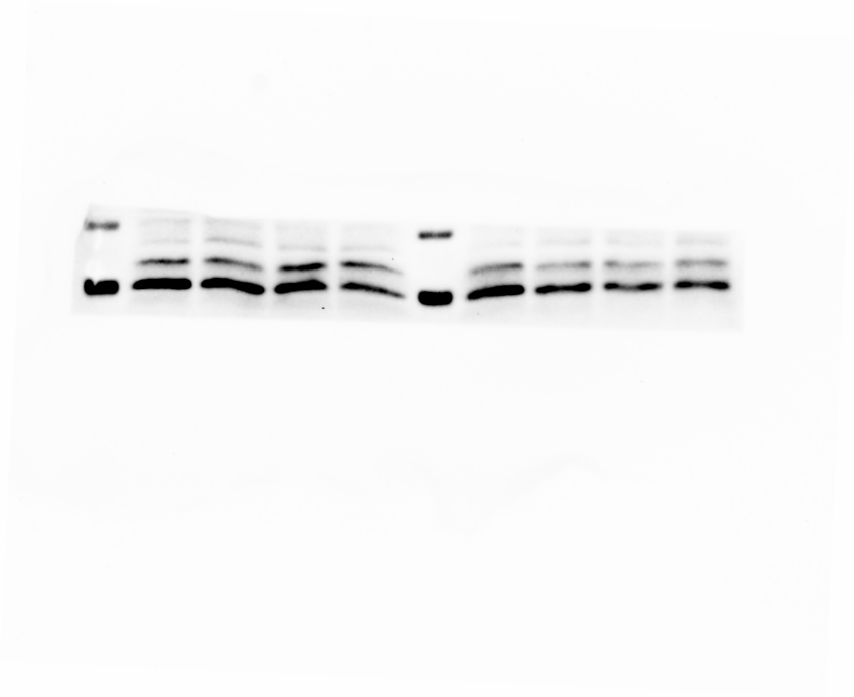

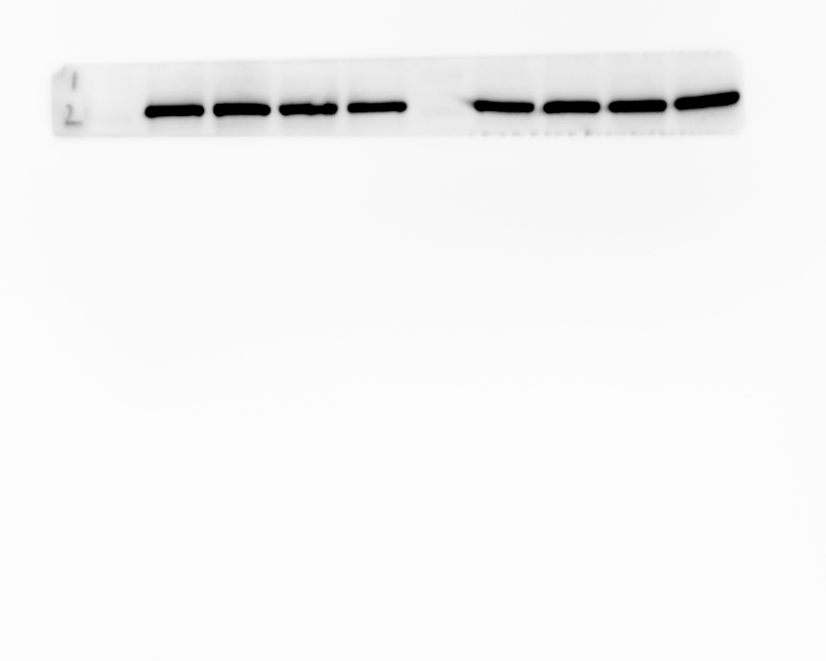


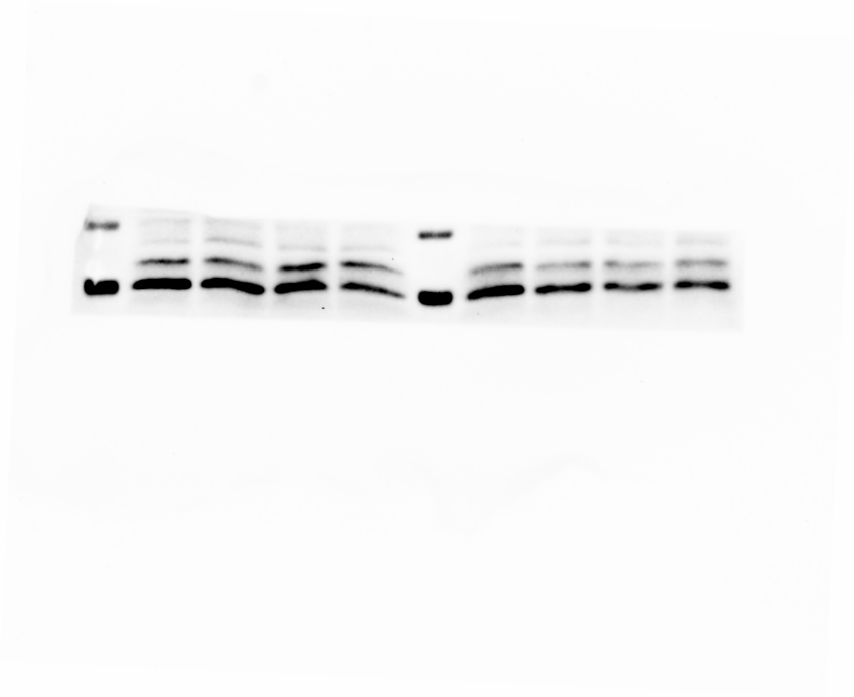

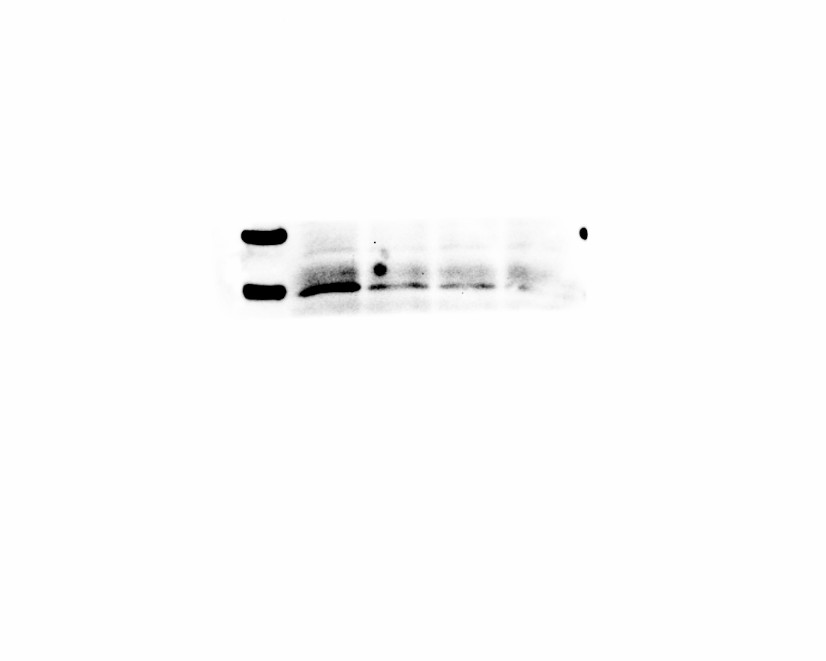

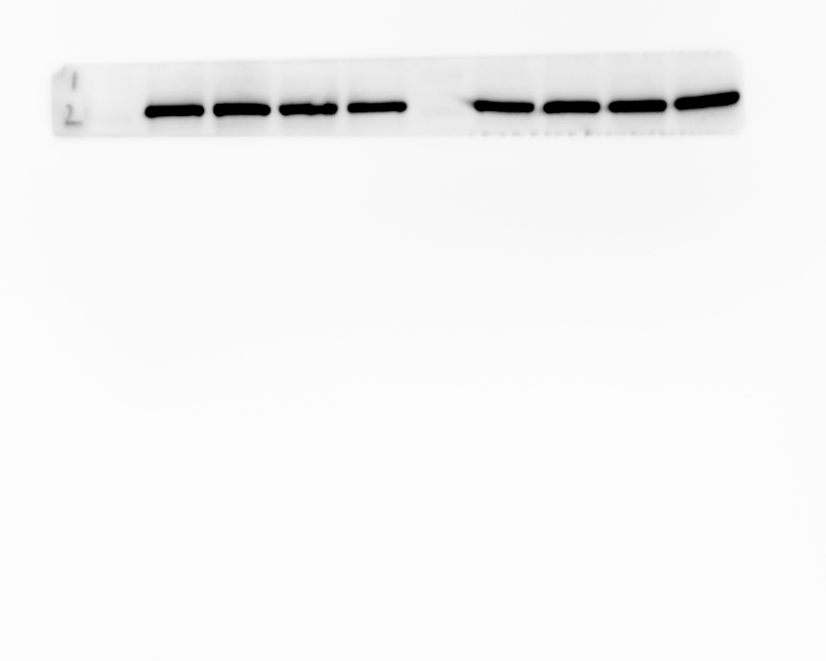


**Figure 5**


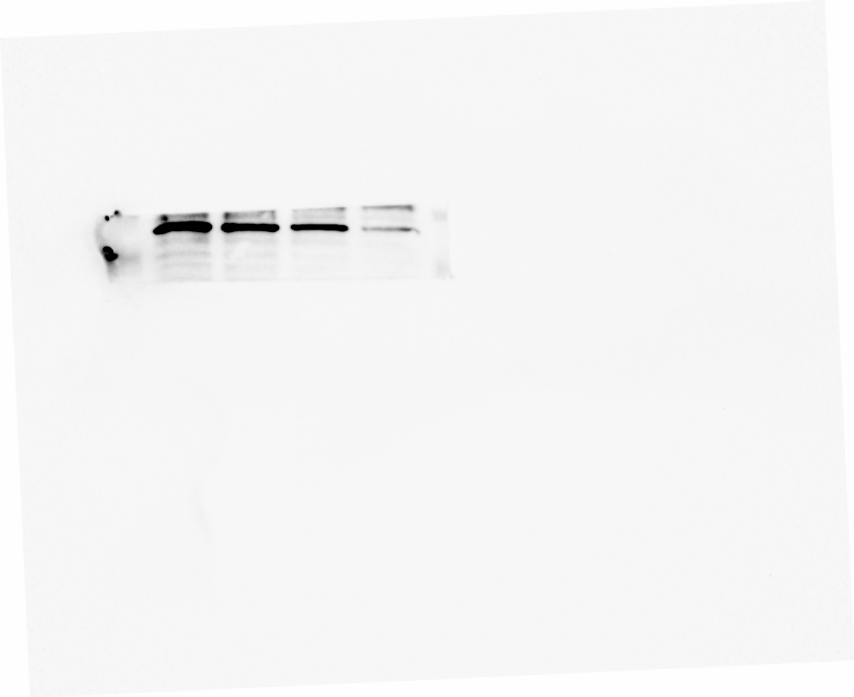

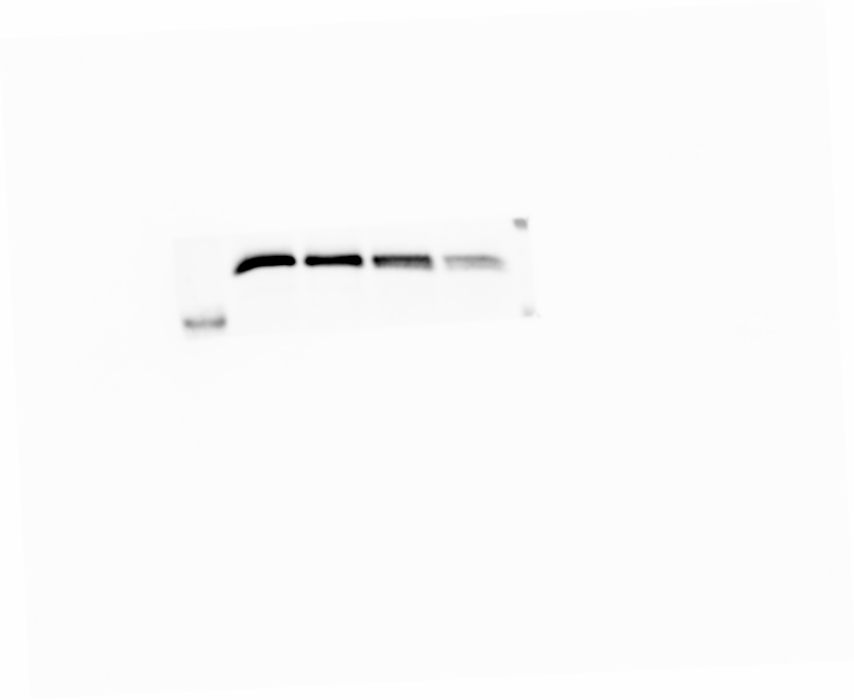

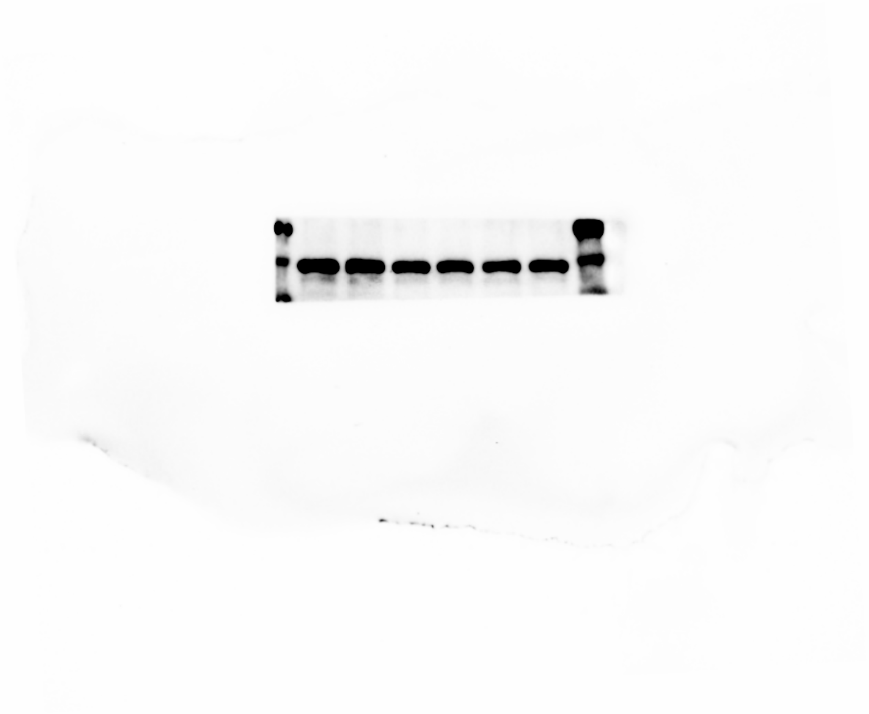

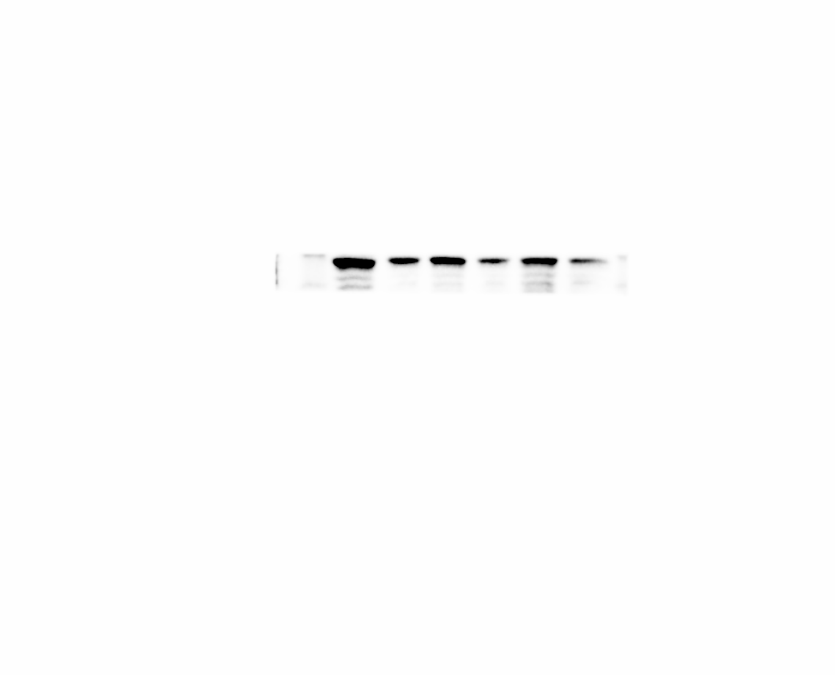

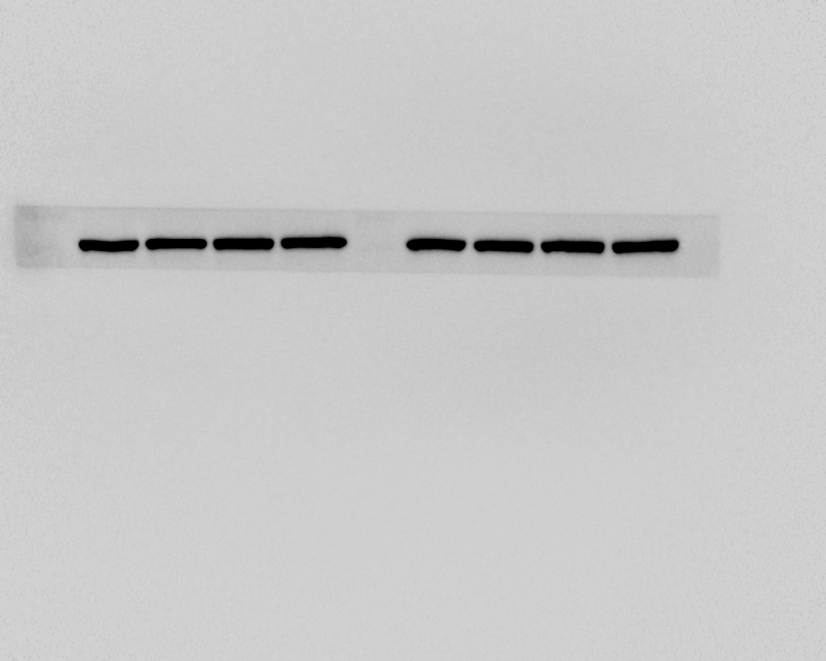


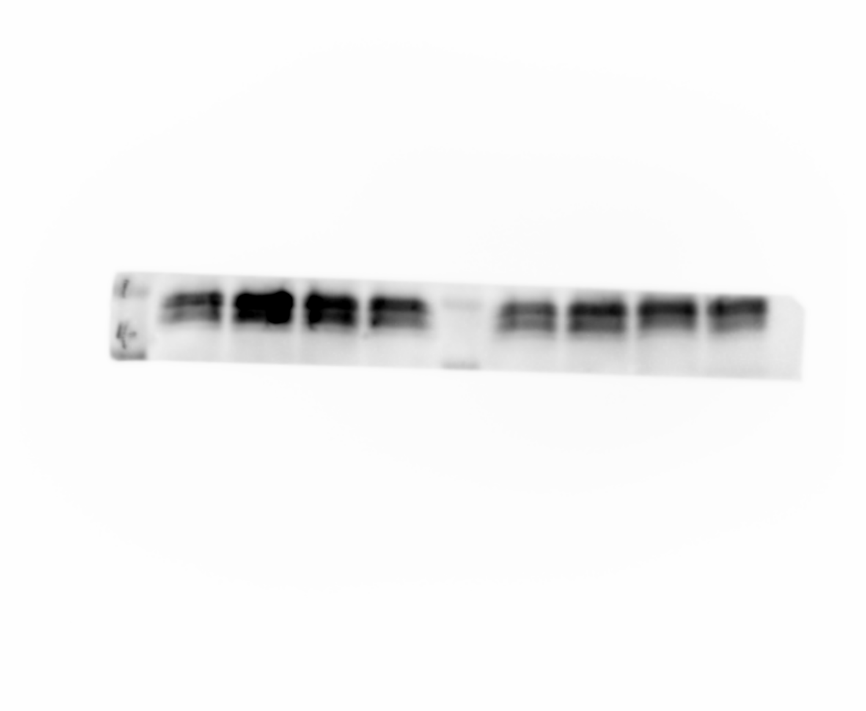

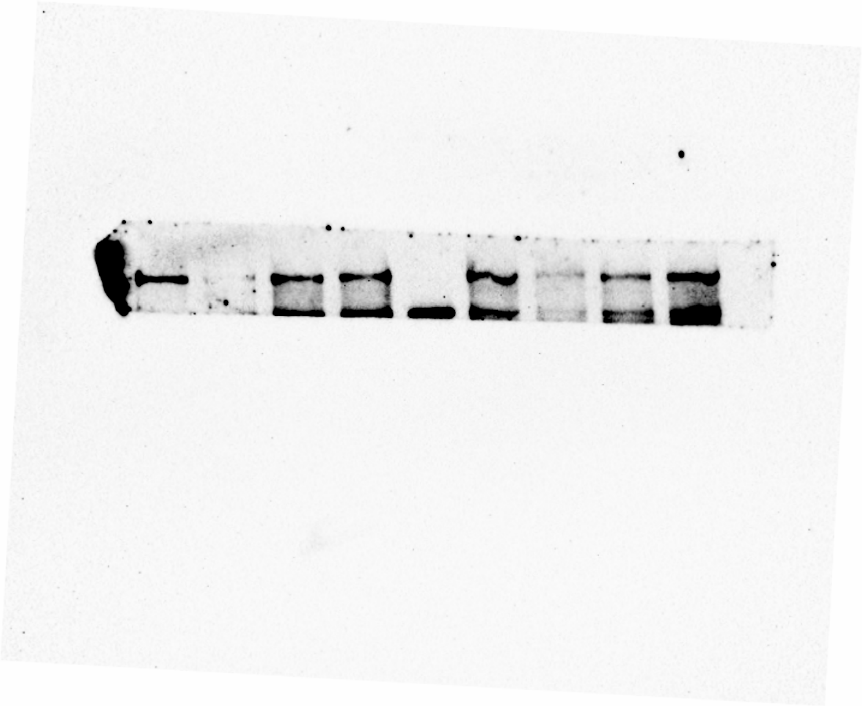

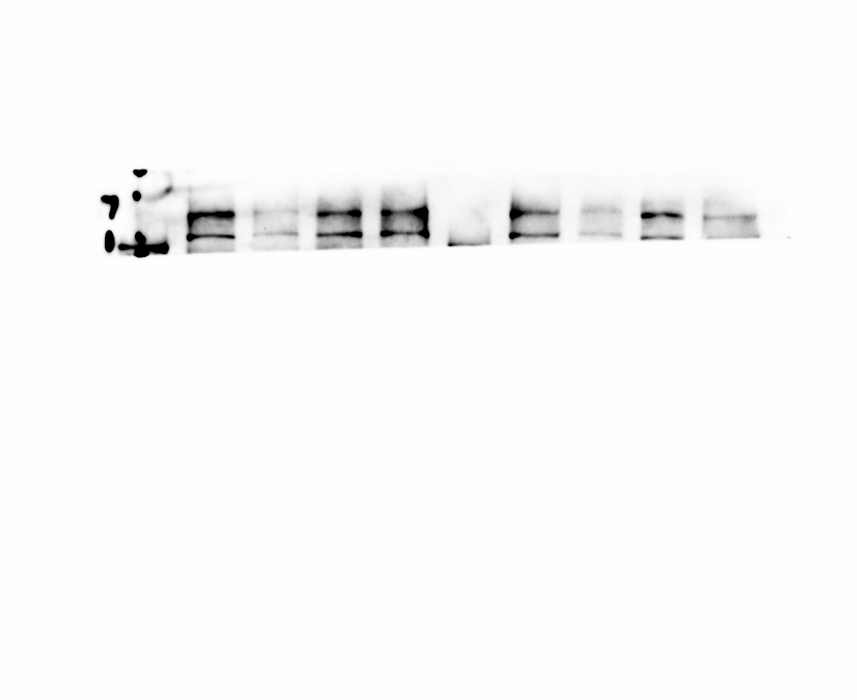

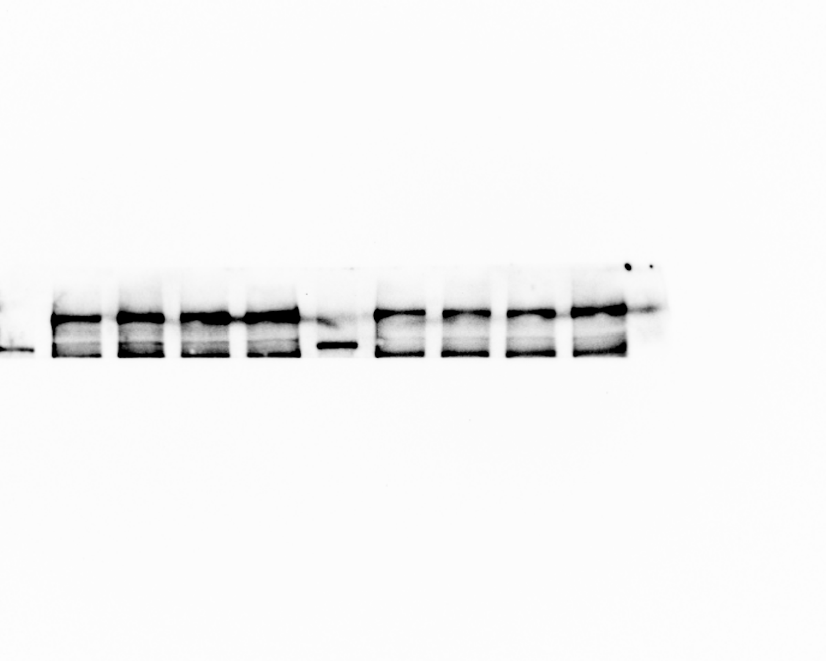

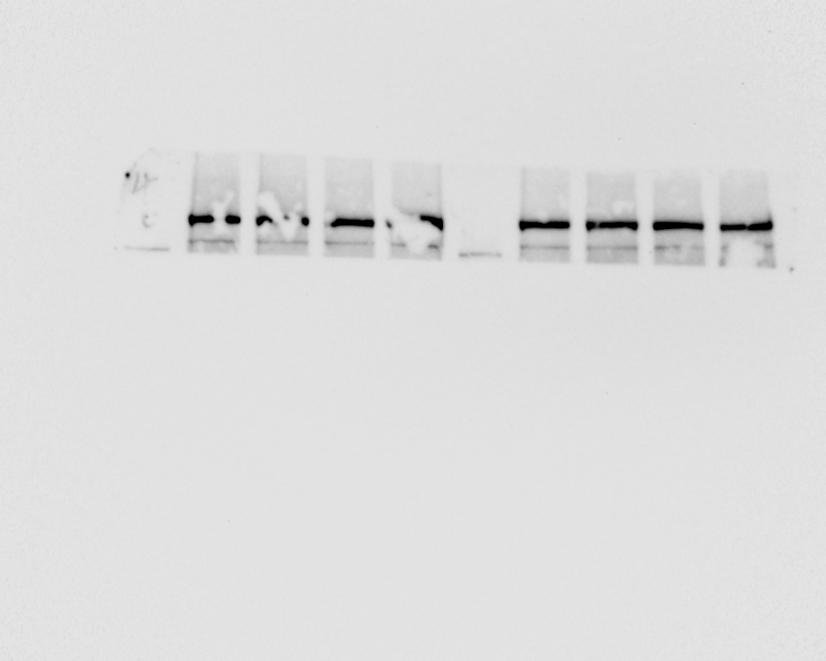

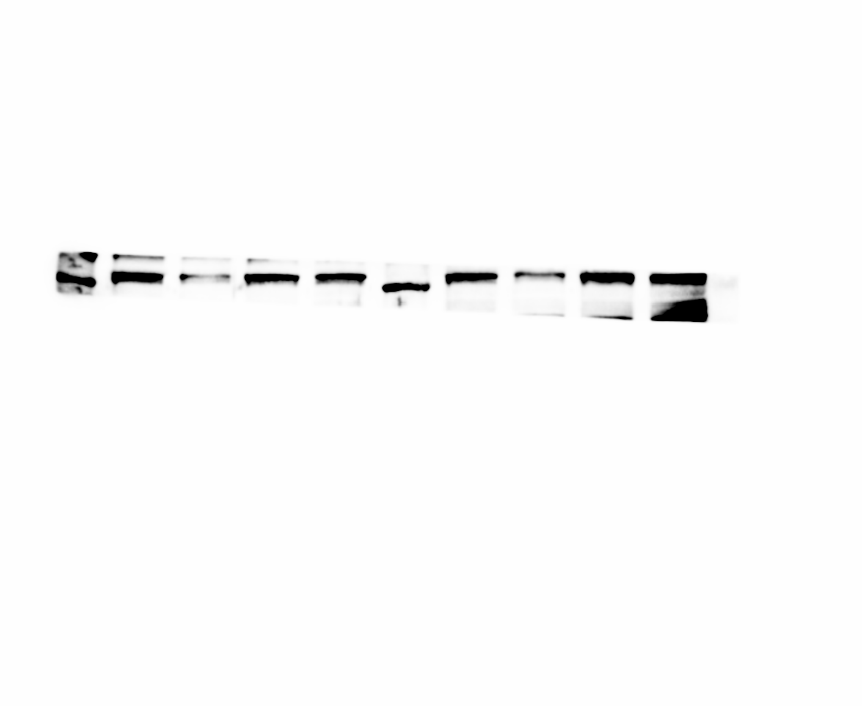

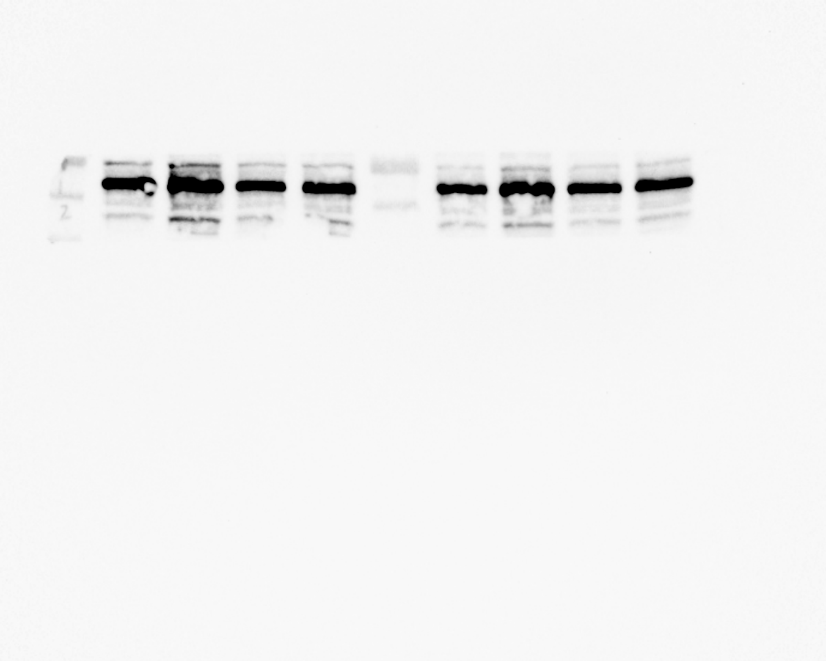

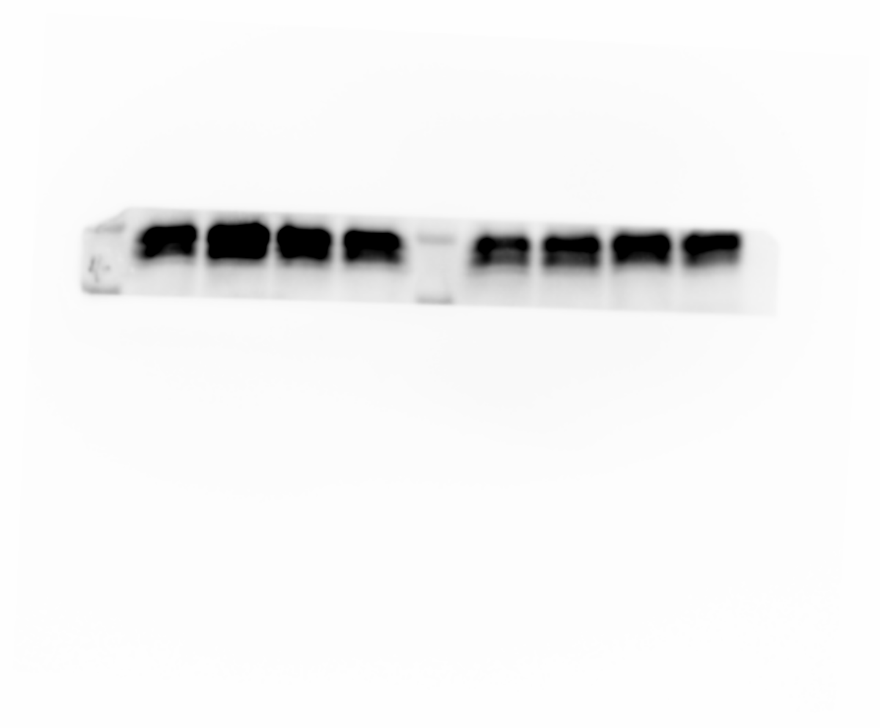


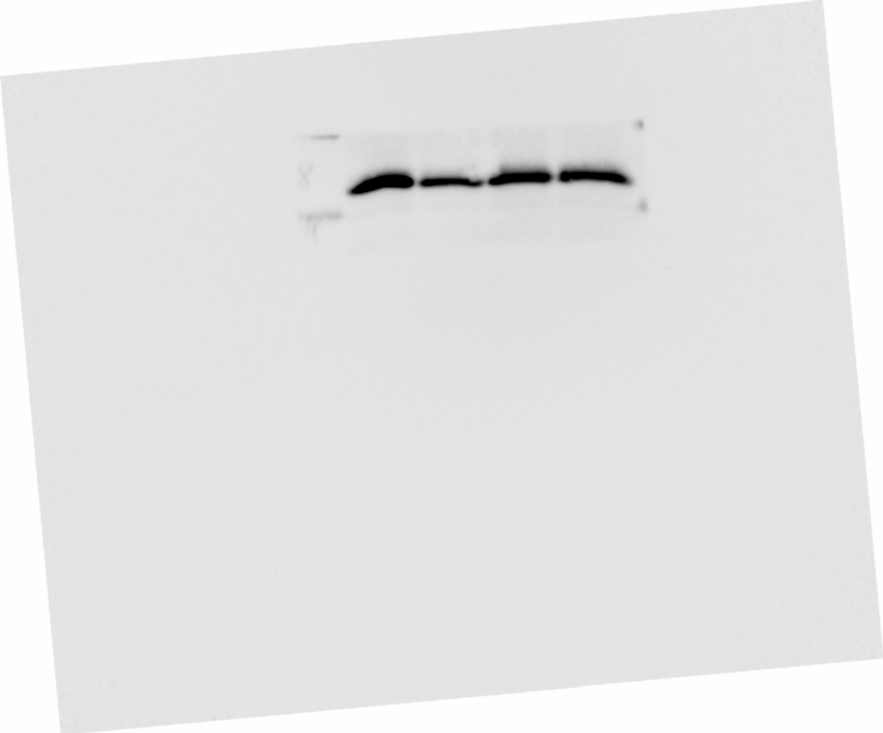

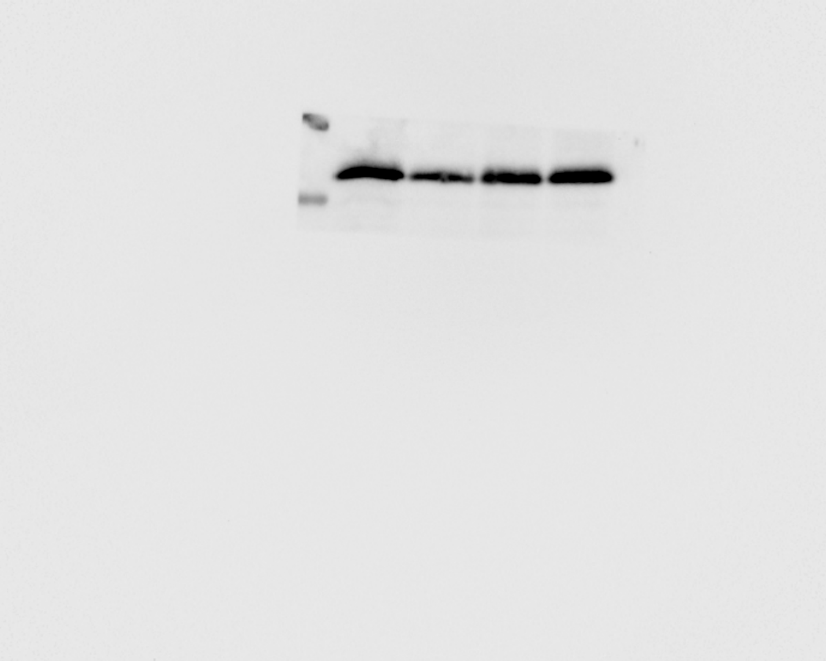

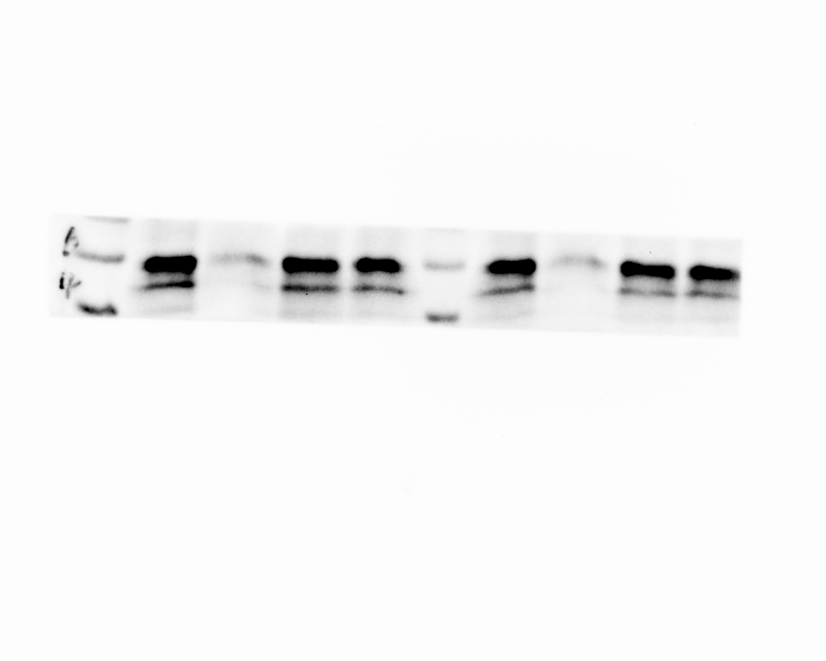

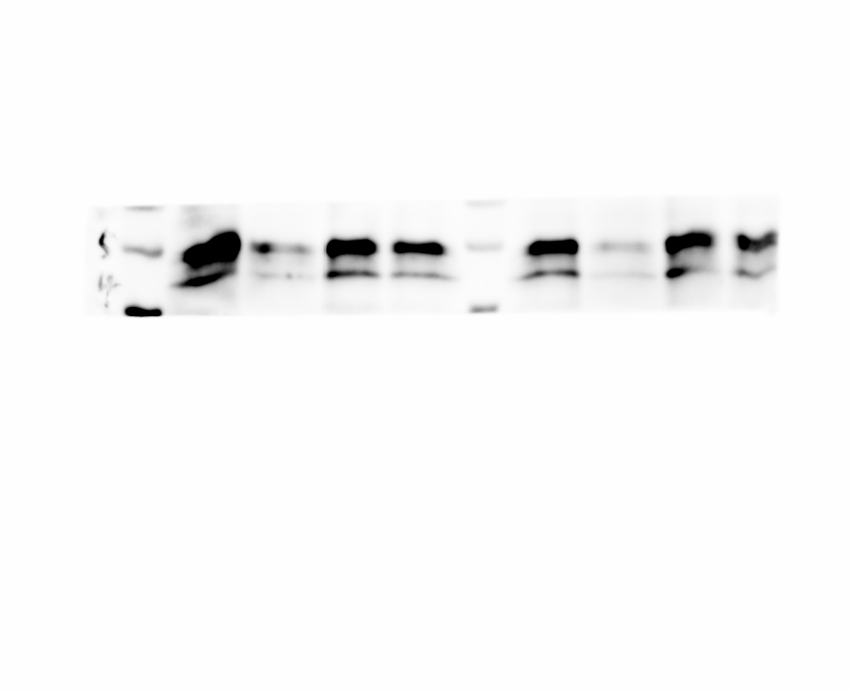

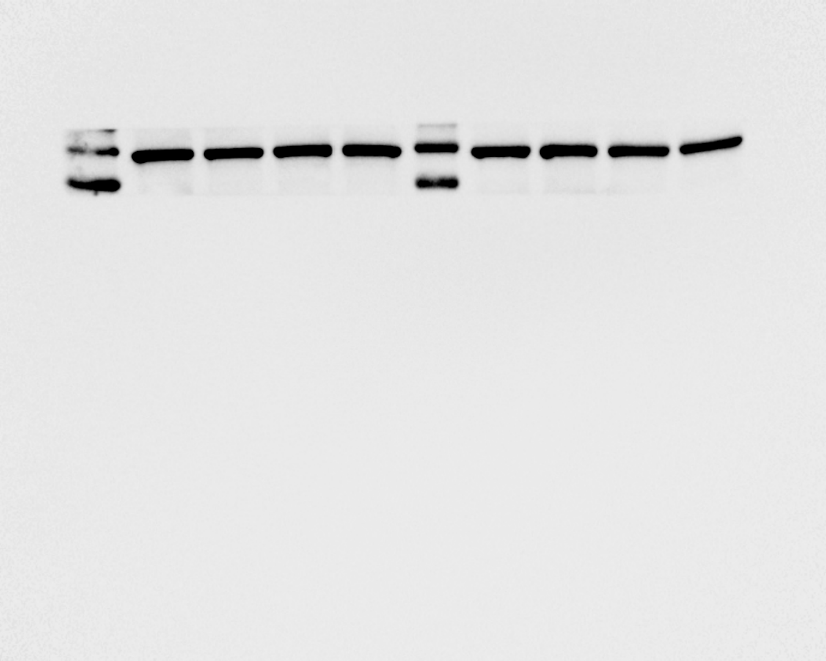


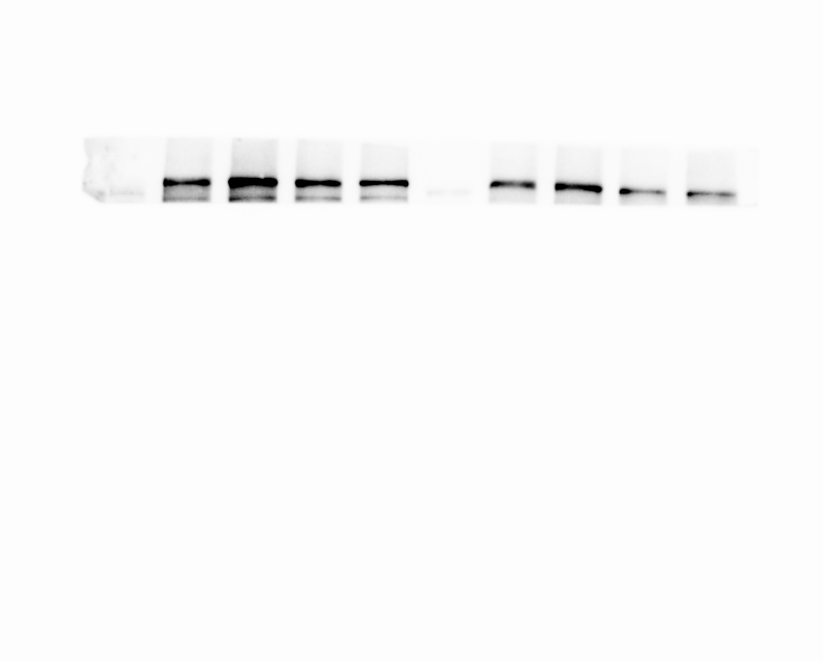

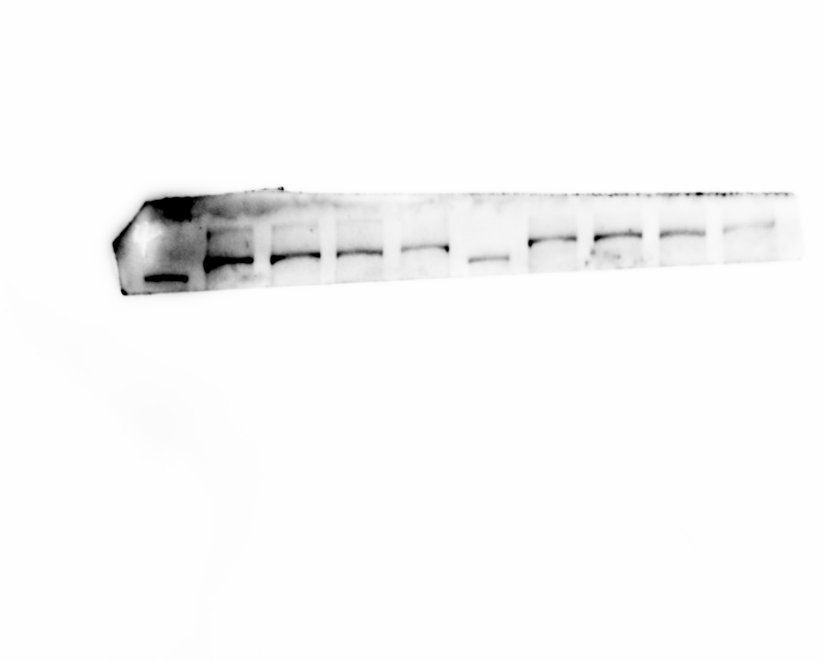

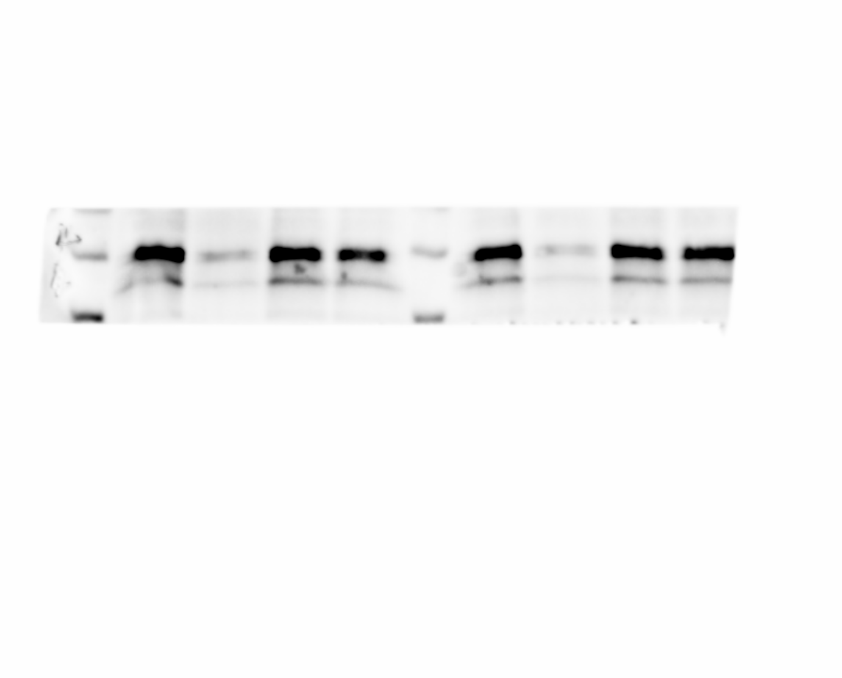

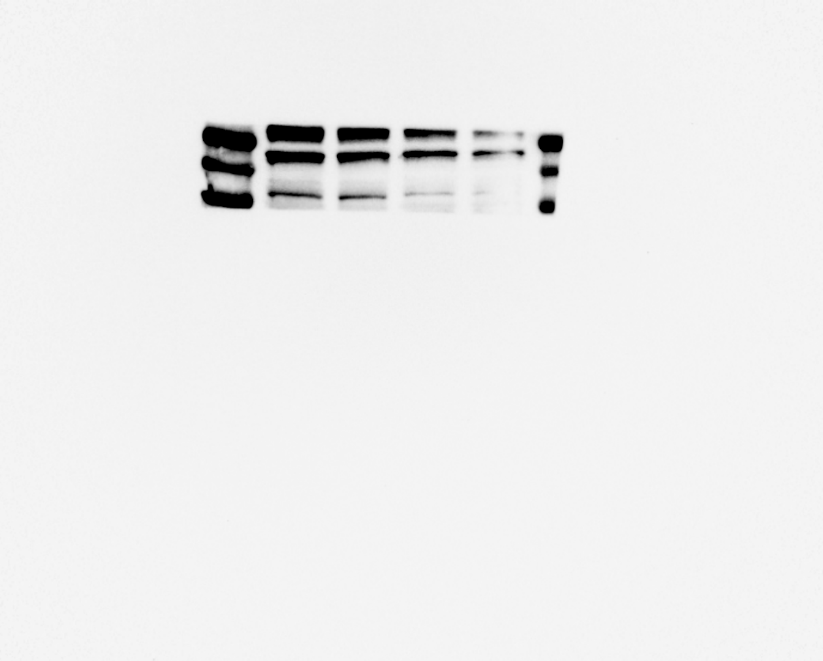

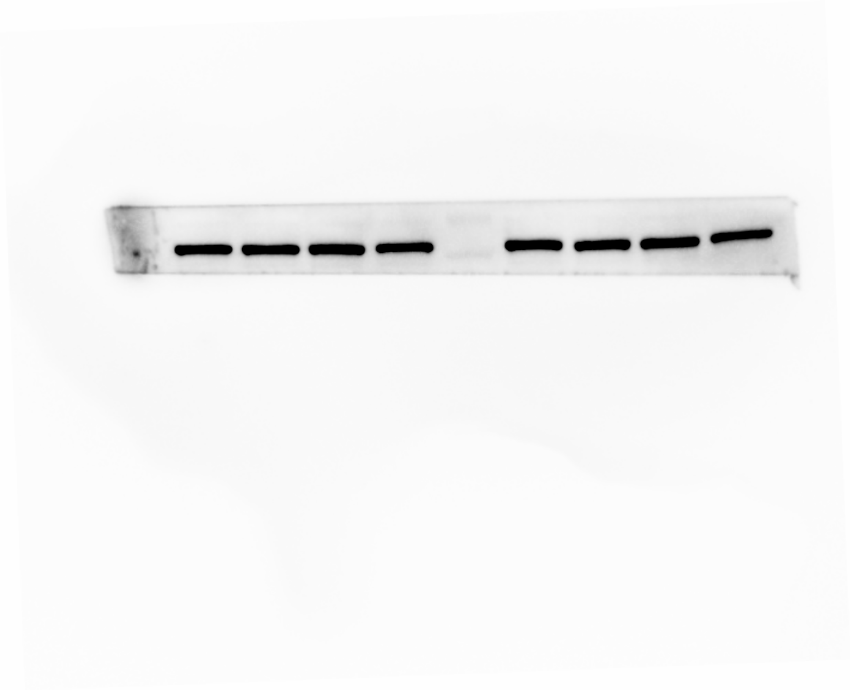

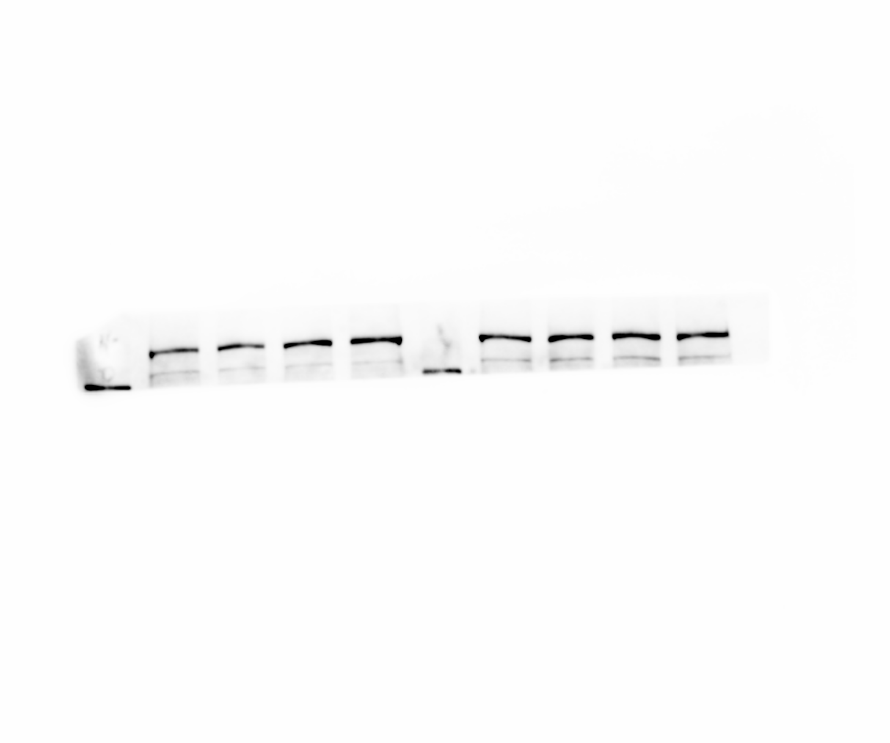

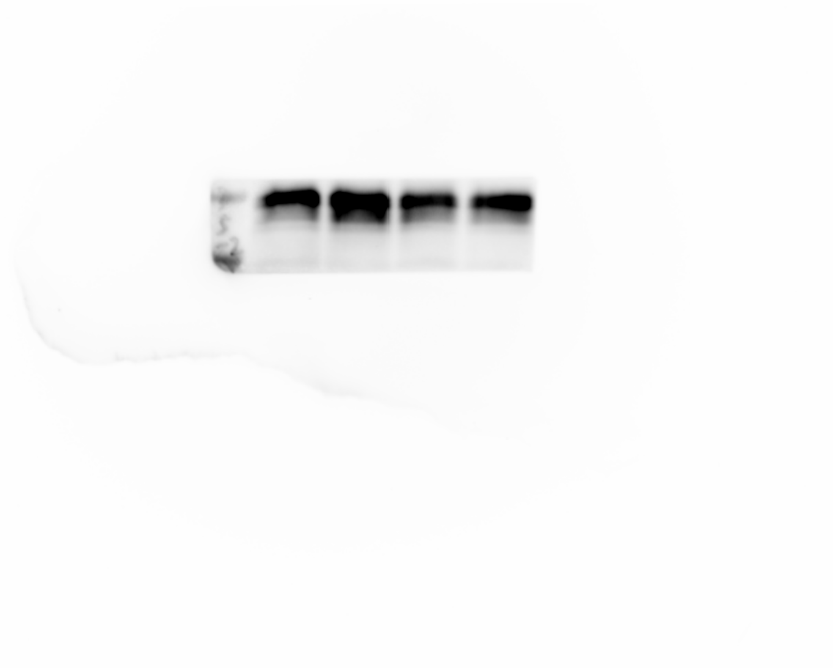

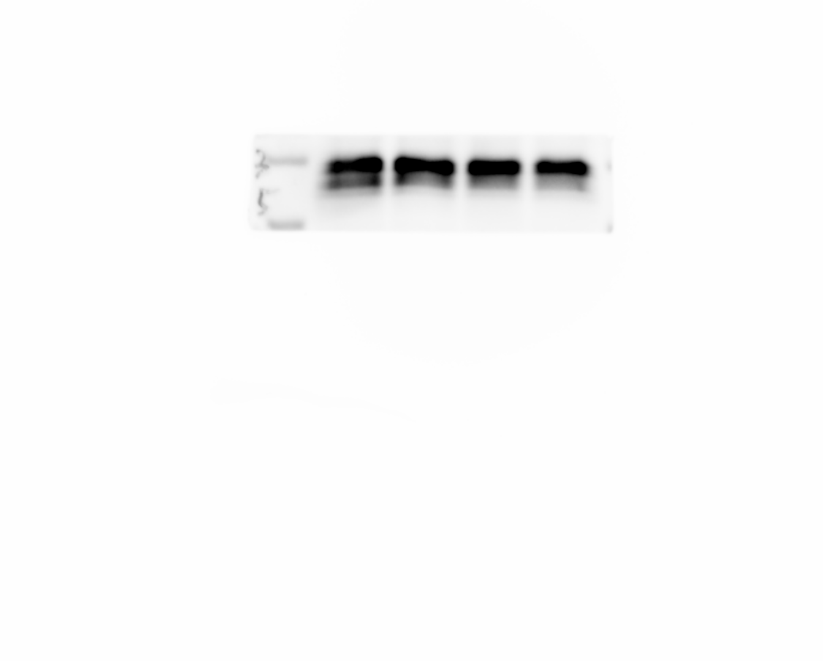

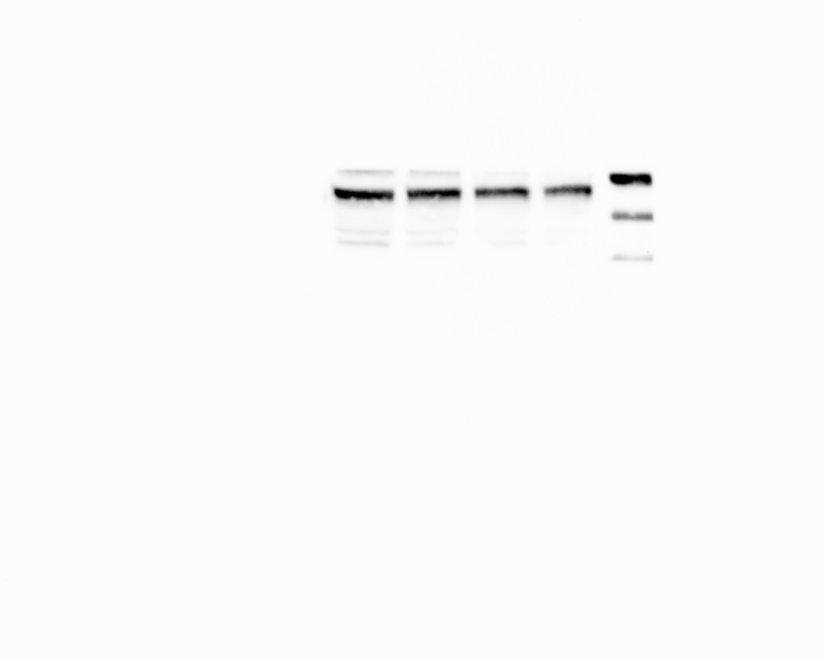

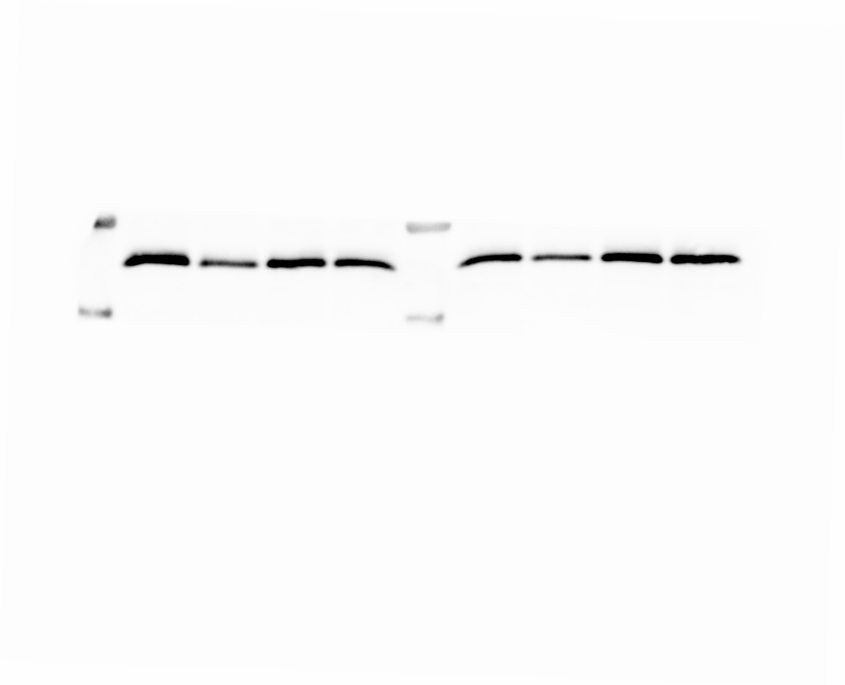

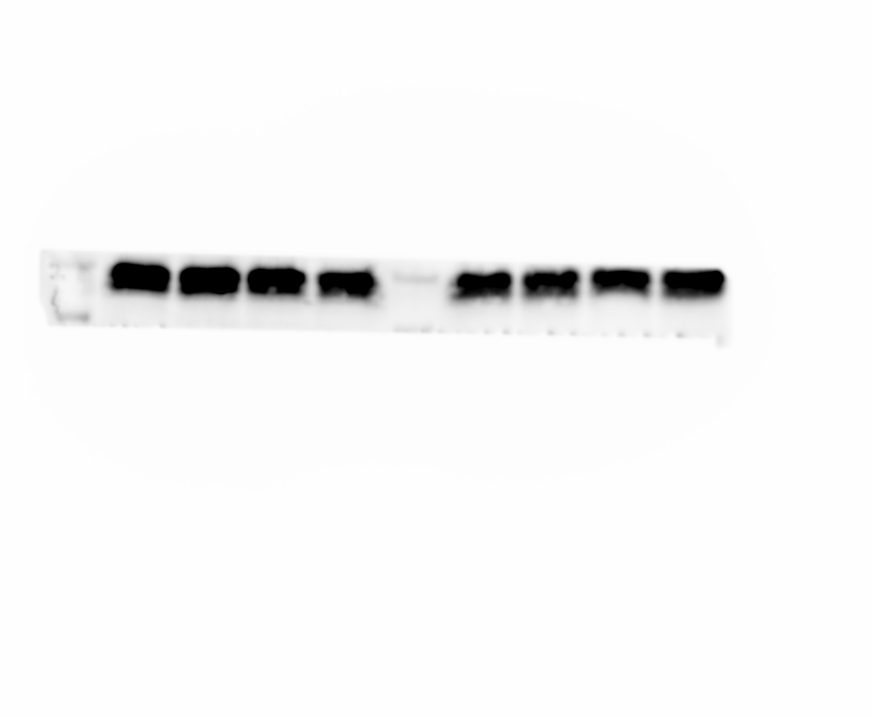

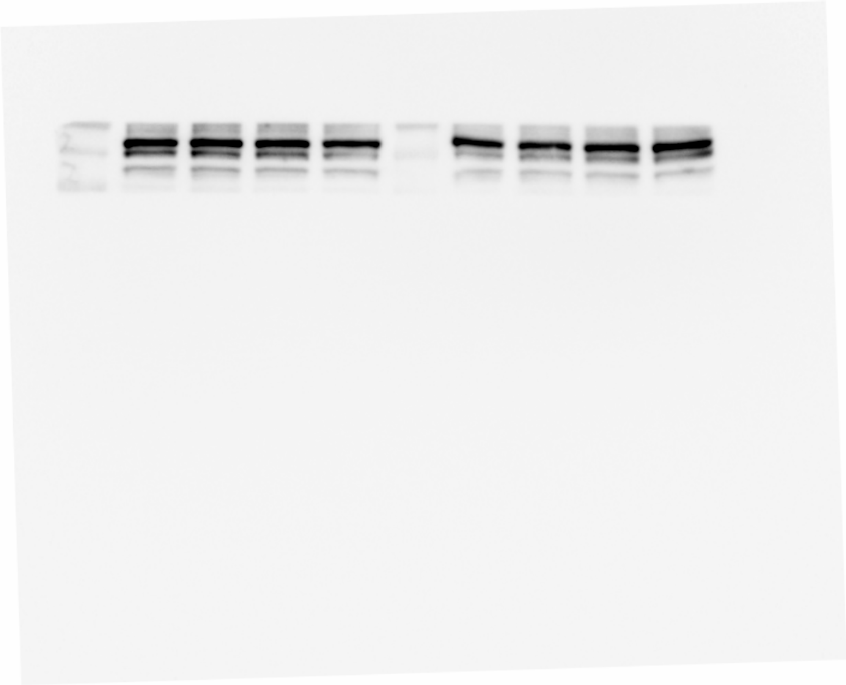

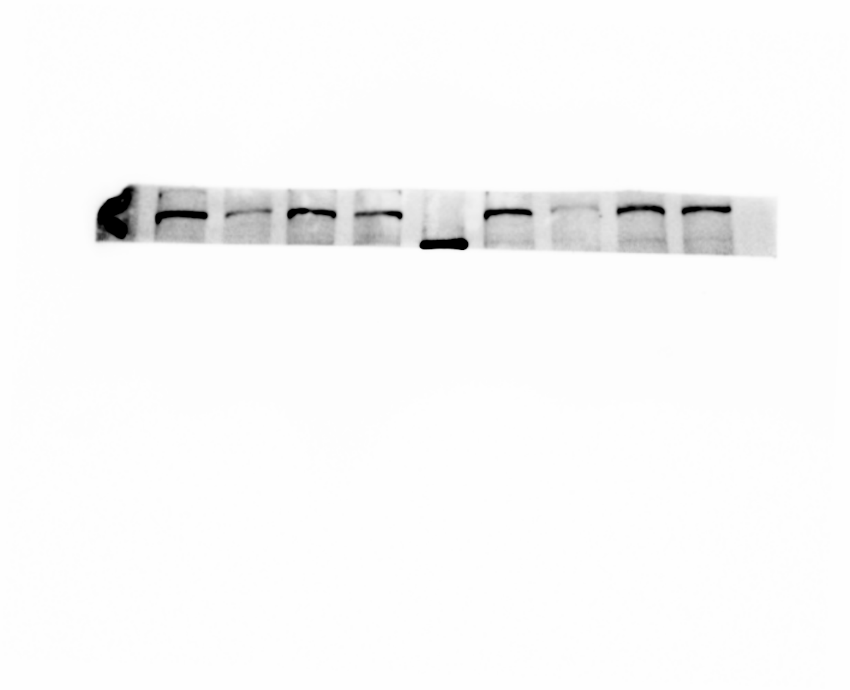

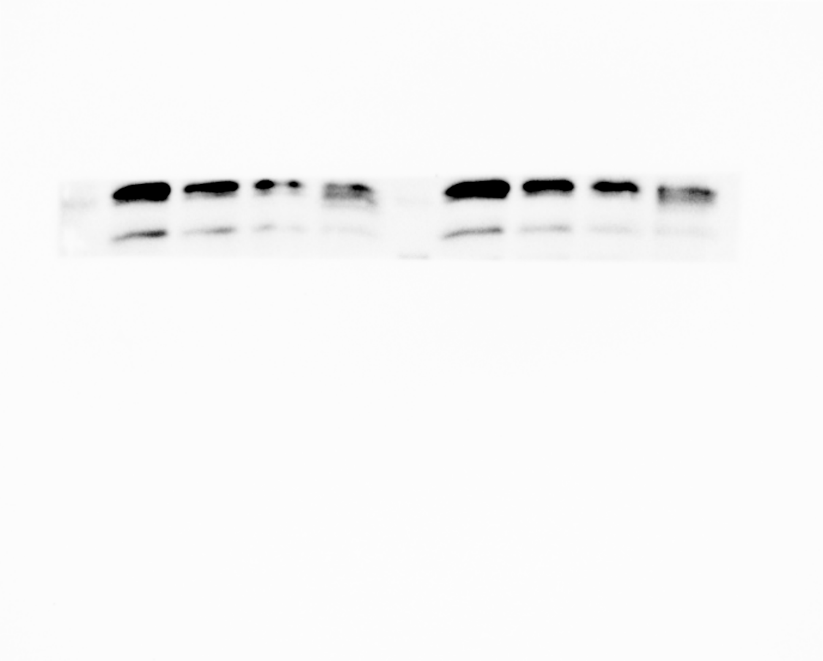

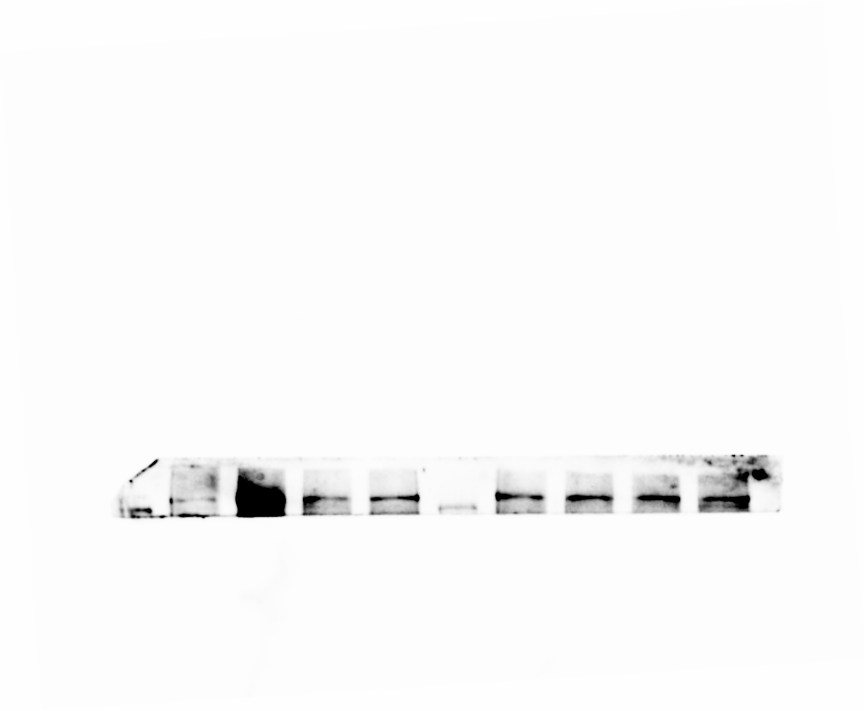

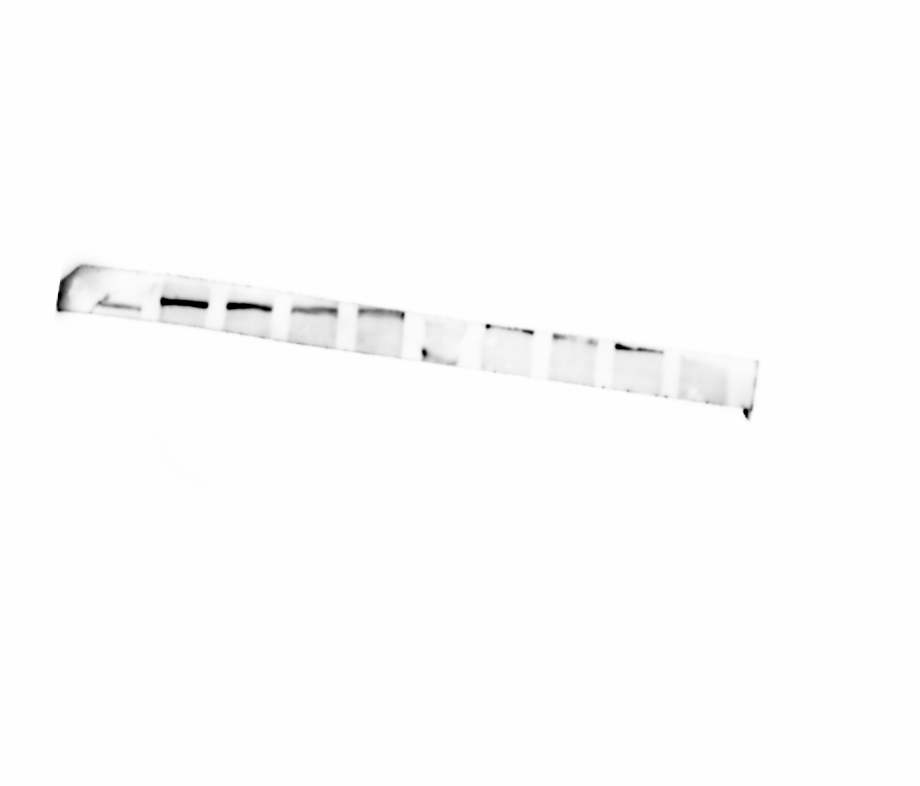

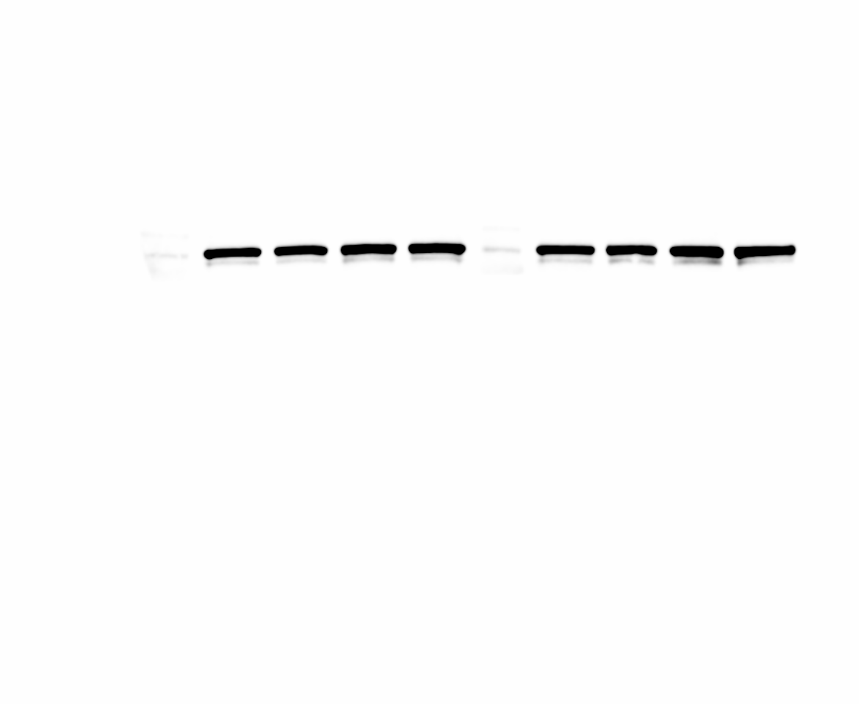


**Figure 6**


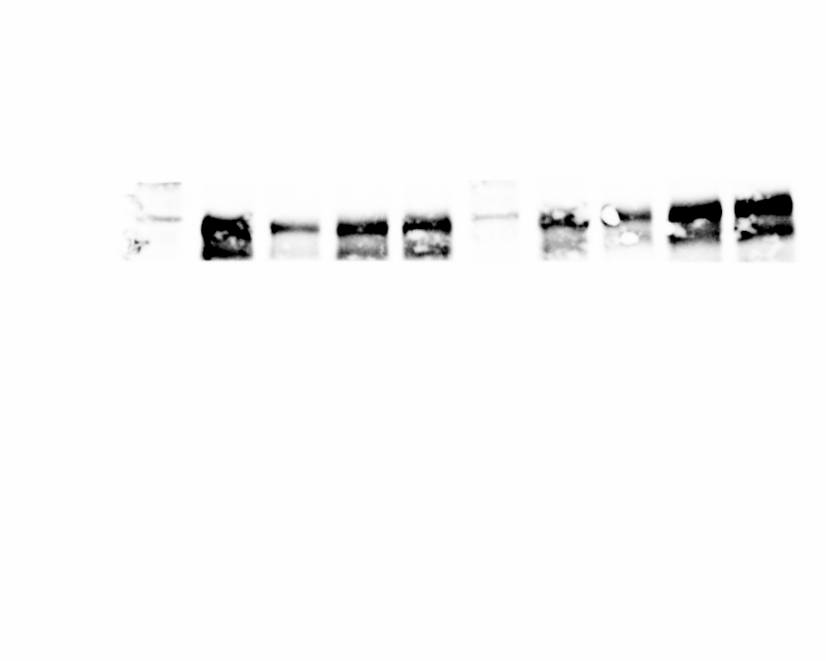

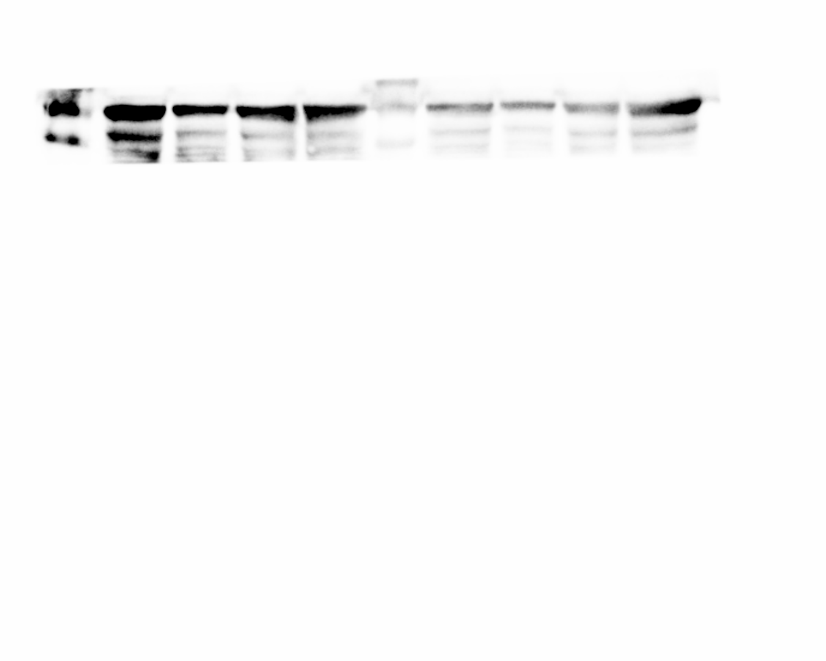

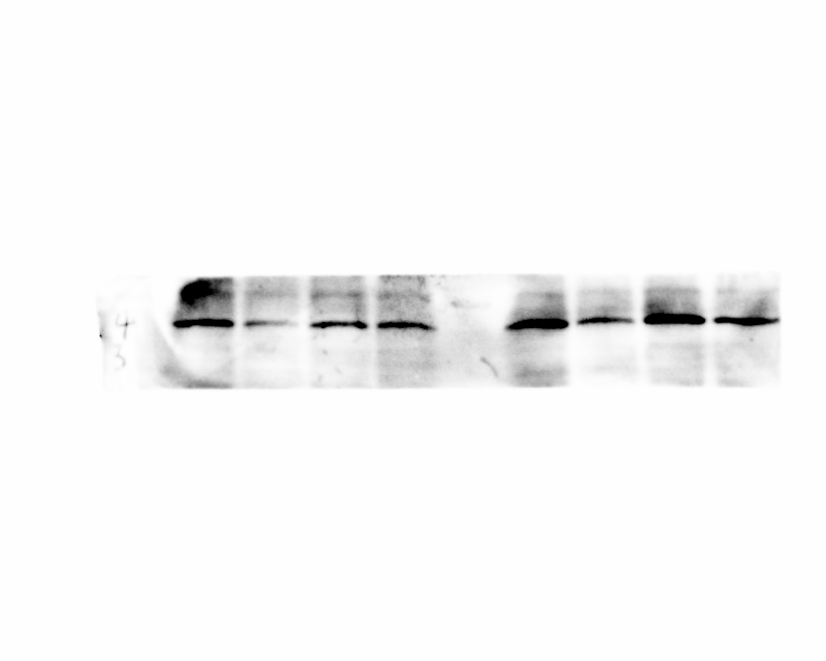

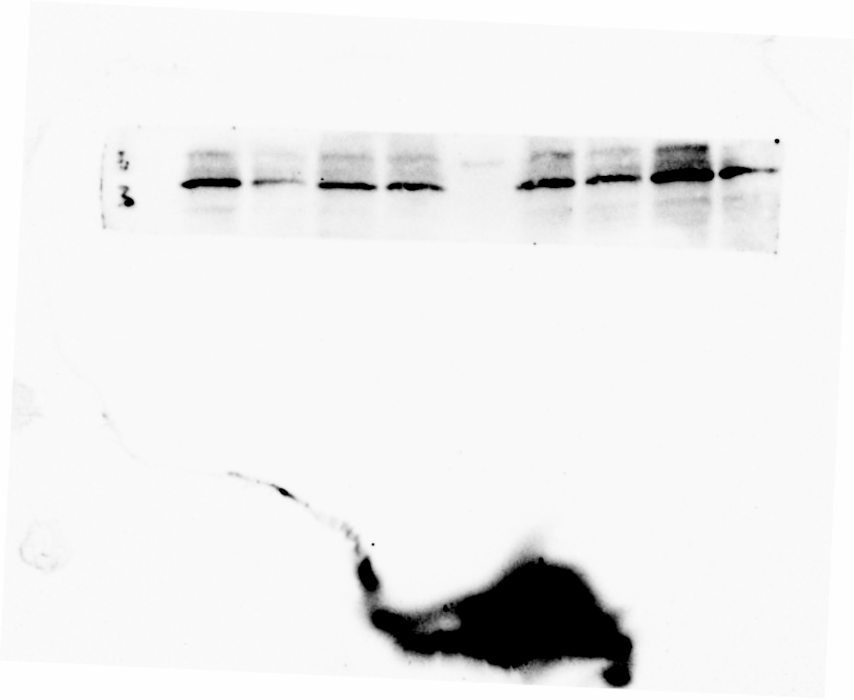

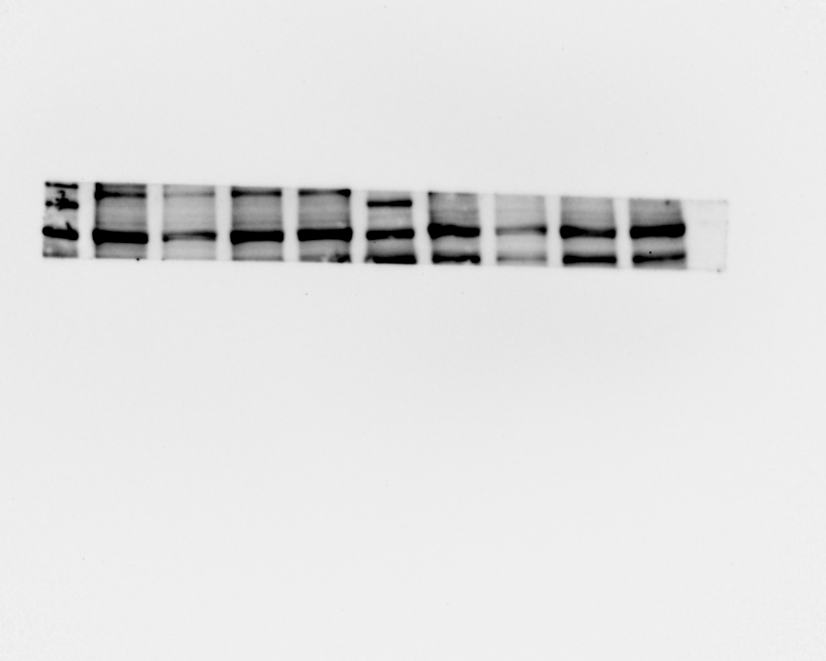

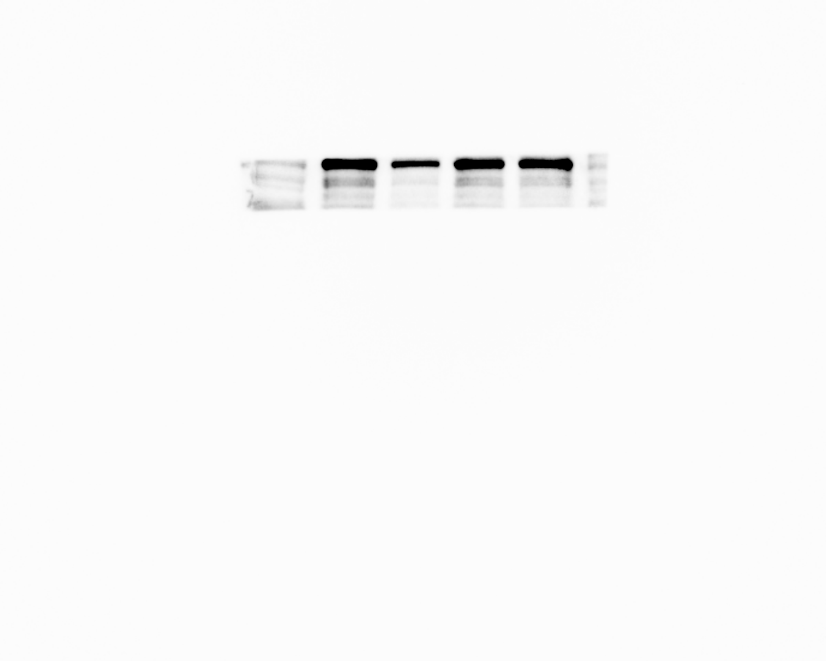

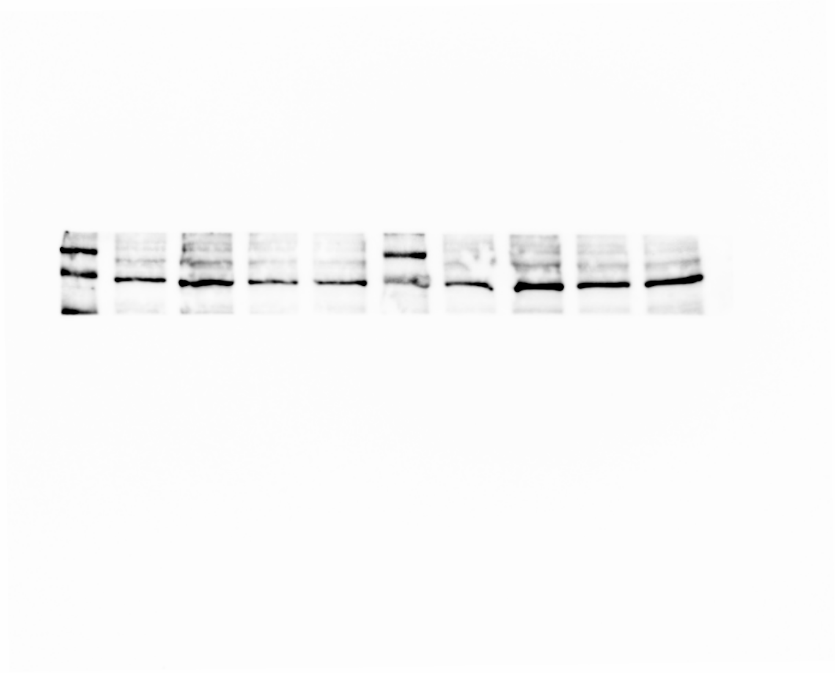

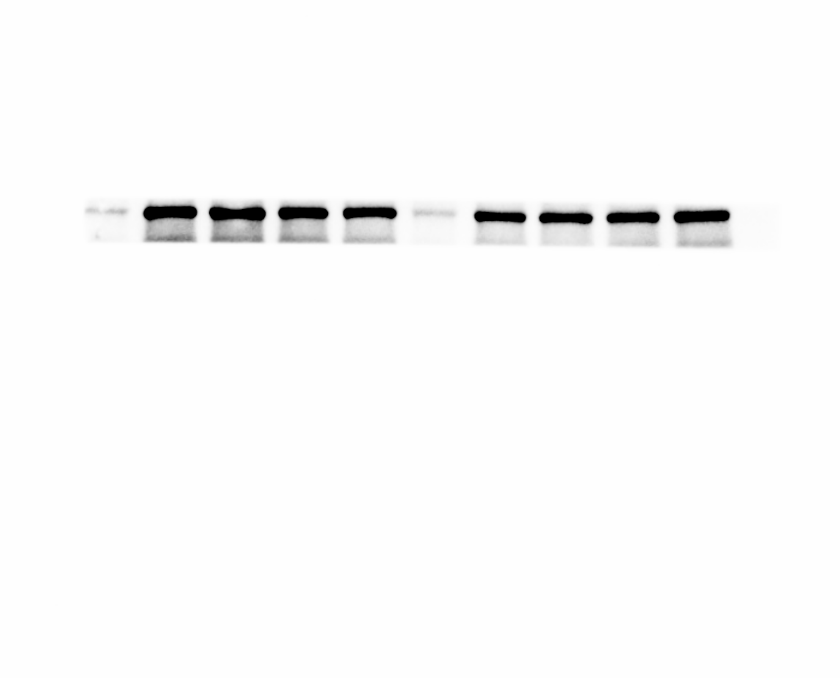

Supplement: Supplementary file 8 — Original Data File [file 41419_2023_5738_MOESM8_ESM.docx]
