# Supplementary material for: Fatostatin induces ferroptosis through inhibition of the AKT/mTORC1/GPX4 signaling pathway in glioblastoma
Source: Cell Death Dis. 2023 Mar 25;14(3):211. doi: 10.1038/s41419-023-05738-8 (PMC10039896; doi:10.1038/s41419-023-05738-8)
Supplement: Supplementary file 6 — AJE Editing Certificate [file 41419_2023_5738_MOESM6_ESM.pdf]

Editing Certificate

This document certifies that the manuscript

Fatostatin induces ferroptosis through inhibition of the AKT/mTORC1/GPX4 signaling pathway in glioblastoma

prepared by the authors

Jiayang Cai 1,2#, Zhang Ye 1,2#, Liguo Ye3, Lun Gao 1,2, Yixuan Wang1,2, Qian sun 1,2, Shiao Tong 1,2, Shenqi Zhang1,2, Liquan Wu1,2, Ji'an Yang 1,2, *, Qianxue Chen 1,2, *

was edited for proper English language, grammar, punctuation, spelling, and overall style by one or more of the highly qualified native English speaking editors at AJE.

This certificate was issued on **February 16, 2023** and may be verified on the AJE website using the verification code **AF16-B429-6DE1-B470-BC16** .

Neither the research content nor the authors' intentions were altered in any way during the editing process. Documents receiving this certification should be English-ready for publication; however, the author has the ability to accept or reject our suggestions and changes. To verify the final AJE edited version, please visit our verification page at aje.com/certificate. If you have any questions or concerns about this edited document, please contact AJE at support@aje.com.

AJE provides a range of editing, translation, and manuscript services for researchers and publishers around the world. For more information about our company, services, and partner discounts, please visit aje.com.
